# Structural fragment clustering reveals novel structural and functional motifs in *α*-helical transmembrane proteins

**DOI:** 10.1186/1471-2105-11-204

**Published:** 2010-04-26

**Authors:** Annalisa Marsico, Andreas Henschel, Christof Winter, Anne Tuukkanen, Boris Vassilev, Kerstin Scheubert, Michael Schroeder

**Affiliations:** 1 Bioinformatics department, Biotechnology Center TU Dresden, Dresden, Germany

## Abstract

**Background:**

A large proportion of an organism's genome encodes for membrane proteins. Membrane proteins are important for many cellular processes, and several diseases can be linked to mutations in them. With the tremendous growth of sequence data, there is an increasing need to reliably identify membrane proteins from sequence, to functionally annotate them, and to correctly predict their topology.

**Results:**

We introduce a technique called structural fragment clustering, which learns sequential motifs from 3D structural fragments. From over 500,000 fragments, we obtain 213 statistically significant, non-redundant, and novel motifs that are highly specific to *α*-helical transmembrane proteins. From these 213 motifs, 58 of them were assigned to function and checked in the scientific literature for a biological assessment. Seventy percent of the motifs are found in co-factor, ligand, and ion binding sites, 30% at protein interaction interfaces, and 12% bind specific lipids such as glycerol or cardiolipins. The vast majority of motifs (94%) appear across evolutionarily unrelated families, highlighting the modularity of functional design in membrane proteins. We describe three novel motifs in detail: (1) a dimer interface motif found in voltage-gated chloride channels, (2) a proton transfer motif found in heme-copper oxidases, and (3) a convergently evolved interface helix motif found in an aspartate symporter, a serine protease, and cytochrome *b*.

**Conclusions:**

Our findings suggest that functional modules exist in membrane proteins, and that they occur in completely different evolutionary contexts and cover different binding sites. Structural fragment clustering allows us to link sequence motifs to function through clusters of structural fragments. The sequence motifs can be applied to identify and characterize membrane proteins in novel genomes.

## Background

Integral membrane proteins play essential roles in living cells by transporting ions and small molecules across the membrane, participating in signal transduction and light harvesting. Although they account for about 20-30% of the open reading frames of various sequenced genomes [1,2], they represent only less than 2% of the Protein Data Bank (PDB), due to the difficulty to obtain high-resolution structures [2,3]. Many disease-linked point mutations, which can lead to misfolding and misfunction [4,5], occur in membrane proteins [6,7].

There has been considerable research in the area of membrane protein structure and function, particularly with respect to sequences, topology, and the effect of mutations [3]. Even though the number of experimentally known membrane protein structures has increased in the last few years, an exhaustive analysis of structural features is still widely needed for enhancing the understanding of many basic phenomena underlying functions, for annotation of large scale genome sequencing data, modeling, and drug design.

Proteins in general are known to be rich in small 3D structural motifs important for protein folding and stability as well as for function [8,9]. Structural motifs are commonly occurring small sections in proteins that can characterise active sites, play a structural role in protein folding, and are involved in enzyme biological functions.

Sequence-structure correlation studies of small structural motifs have been carried out and several motif databases have been developed in the past few years [10-12], including the I-sites library, developed by Baker and co-workers [10] and the MSDmotif database at the EBI, developed from Thornton and co-workers [13]. Most of the documented 3D motifs show unique patterns of hydrogen bonds, patterns of highly conserved residues, and particular distributions of backbone torsion angles.

When it is possible to associate sequence patterns with structural motifs, they can be used to predict the occurrence of the motifs in new sequences to improve structure prediction methods or help functional annotation such as Prosite [14] or ProFunc [15].

Although the structural roles of several small 3D motifs have been widely recognized, their functional roles are not always known. Numerous experiments demonstrate the important role played by helix caps in stabilizing helical termini [16], and linking secondary/supersecondary structure elements. In some cases, structural motifs have been found to be functionally very important: beta-hairpins in specific protein-protein interactions [17] and nest motifs, as part of small hydrogen-bonded motifs, are prominent in P-loops [9].

Integral *α*-helical membrane proteins are composed of a bundle of *α*-helices that completely span the membrane. Besides motifs that are also common to globular proteins, *α*-helical transmembrane proteins are rich in reentrant regions [18], interfacial helices [19], irregular structures at the water-membrane interface, and structured extracellular or cytoplasmic loops [3]. Furthermore, helix-helix interaction motifs have been defined by Walters and co-workers [20] by few clusters of 3D helical pairs in transmembrane proteins. Among these motifs, the most important are the GAS_*left *_and GAS_*right *_motifs, characterized by high propensities of the small residues Gly, Ala and Ser to occur at periodic positions in the helix-helix interfaces. Although these transmembrane protein structure-sequence motifs are very important from a functional point of view [18,19,21], very few motif databases focus on transmembrane proteins. There are no comprehensive studies, to our knowledge, that focus on functional/structural motifs in transmembrane proteins. Among recent studies on transmembrane protein motifs, the TOPDOM database [22] collects domains and transmembrane protein sequence motifs from different motif databases and organizes them by their location in the protein with respect to the lipid layer.

In the present work we describe three novel motifs in transmembrane proteins and a novel computational approach, structural fragment clustering, which learns sequential motifs from 3D structural fragments. The motifs are a dimer interface motif in voltage-gated chloride channels, a proton transfer motif in heme-copper oxidases and a convergently evolved interface helix motif in aspartate symporter, serine protease and cytochrome *b*. These motifs were chosen from among a list of 58 novel motifs specific to transmembrane proteins, because, besides being statistically significant and novel with respect to the Prosite database, they are filtered on the basis of an accurate functional annotation and manual checking in the scientific literature. Furthermore, the chosen motifs are biologically significant as they play an important role in elucidating the protein functions of specific families or they evolved independently and occur in different families, *i.e*. convergent evolution.

Only a few fragment-based clustering methods exist that can automatically identify motifs and relate them to function [23]. These methods are based either on geometric features of the fragments or secondary structure patterns [9,23,24]. In another study, Espalader and co-workers identified loop motifs in proteins associated with specific functions by using the Gene Ontology function [25]. In a recent study [26], Karuppasamy and co-workers used a clustering algorithm based on backbone torsion angles to find fragment clusters enriched in Gene Ontology function and associated with a significant biochemical function. In this study, we cluster transmembrane protein fragments based on common structural features in order to generate a library of linear sequence motifs. New structural motifs and their corresponding signals at sequence level are derived. Our analysis concentrates on *α*-helical transmembrane proteins as they are more abundant in the PDB (2525 protein chains in PDBTM as of August 17, 2007) than beta-barrels (218 protein chains in PBDTM as of August 17, 2007). The identified motifs are described in terms of sequence patterns (regular expressions), structural features, and functional relevance. Three of the most interesting motifs are discussed in detail.

## Results and Discussion

### Biological potential of novel sequence motifs

Three biologically interesting motifs from our library are discussed in the following section. A motif is considered biologically significant if it adds further information, at a detailed structural level, that can help to enhance the understanding of protein function or shed light on mechanisms of structural stability. The chosen motifs are statistically significant, novel with respect to the Prosite database and have very low false positive rates. New functional insights of these novel motifs are assessed by detailed automated functional annotation and manual literature check (see Materials and Methods). Furthermore, the three examples presented below could not be identified by other structure-based methods such as ProFunc [15] or TOPDOM [22]. A list of 58 non-redundant significant novel motifs, their specificity for membrane proteins and basic structural/functional annotation is provided in Additional file 1. These 58 motifs exhibit different structural features and locations with respect to the bilayer planes. Most of them (44%) are regular helix motifs embedded in the hydrophobic membrane core. About 32% are irregular helices or loops at the membrane-water interface, and among them, 20% are short helices parallel to the membrane planes. Only 8% of the motifs are structured loops located on the cytoplasmic or extracellular side with respect to the membrane. About 6% of the motifs form reentrant loops. Other structural features associated to the motifs are: helix kinks, tilted helices, and *π*-bulges in 17% of the cases.

About 33% of the motifs occur in different families, *i.e*. they are either structurally important or independently evolved motifs. About 67% of the motifs seem to be associated with the function of a specific family: 53% of the family-specific motifs belong to Cytochrome b (Pfam: PF00033). The other families covered by the motifs are: cytochrome C and Quinol oxidases (Pfam: PF00115) in 12% of the cases, Bacterial opsins (Pfam: PF01036) in 12% of the cases, Photosynthetic reaction center (Pfam: PF00124) in 9% of the cases and, in few cases, Voltage-gated chloride channels (Pfam: PF00654), ammonium transporters (Pfam: PF00101), NADH-dehydrogenase (PF00146) and G-protein coupled receptor-like superfamily (CL0192). From the functional annotation, it is worth to notice that about 70% of the motifs are found in cofactor/ligand/ion binding sites, suggesting that they are specific for the protein's function. About 30% of the motifs are also found at protein-protein interaction interfaces of transmembrane complexes. Finally, 12% of the motifs are found to bind special kinds of lipids such as glycerol or cardiolipins, which are know to modulate protein function via specific protein-lipid interactions.

#### A- [AS]- [FIV]- [NR]-A-P-L- [AT]-G: a novel dimer interface motif in voltage-gated chloride channels

**Figure 1:**
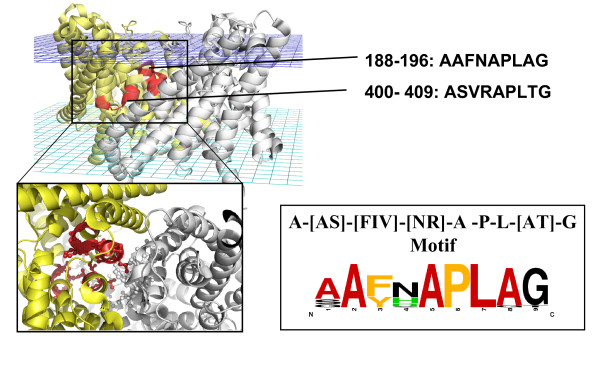
**A- [AS]- [FIV]- [NR]-A-P-L- [AT]-G motif**. Voltage-gated chloride channel, dimeric form (PDB ID: 1kpl, chain: B). The two reentrant regions where the A- [AS]- [FIV]- [NR]-A-P-L- [AT]-G motif is found are shown in red. The sequences of the two reentrant regions are also shown. A multiple sequence alignment of several bacterial chloride channels (derived from a PSI-BLAST search against Swiss-Prot with the 1kpl sequence, data not shown) shows that A- [AS]- [FIV]- [NR]-A-P-L- [AT]-G motif is well conserved across different bacteria (see Logo in the picture), indicating its possible functional implication. At the bottom: a zoomed-in view of a portion of the voltage-gated chloride channel dimerization interface. The residues belonging to the reentrant regions and part of the A- [AS]- [FIV]- [NR]-A-P-L- [AT]-G motif are highlighted in red. The interface residues that are less that 5 Å apart from the red residues are shown in gray.

**Figure 2:**
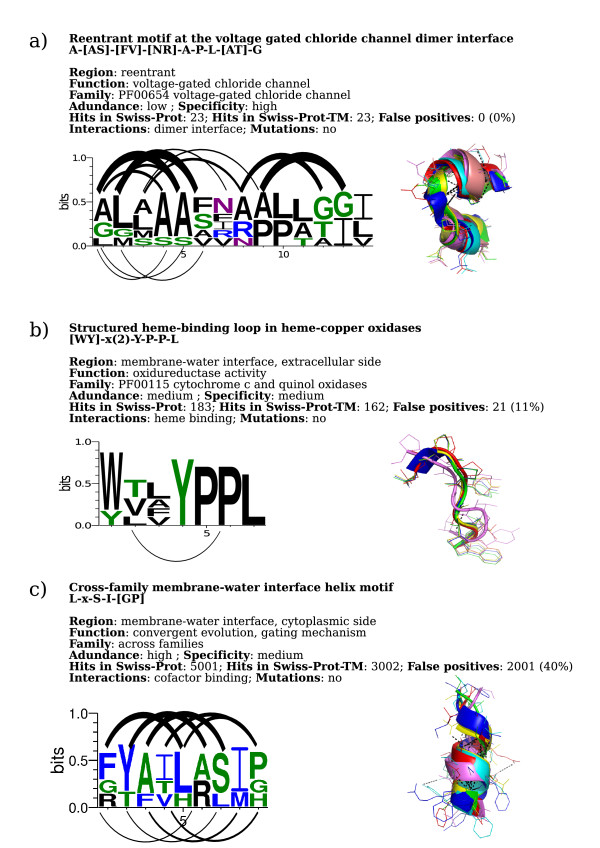
**Three novel biologically significant sequence motifs**. Basic statistics and structural/functional annotation for the **a) **A- [AS]- [FIV]- [NR]-A-P-L- [AT]-G-I motif; **b) **[WY]-x(2)-Y-P-P-L motif and **c) **L-x-S-I- [GP] motif. The *Abundance *field refers to the number of motif hits in the transmembrane proteins in the Swiss-Prot database: a number less than 100 is considered as *low*, between 100 and 500 as *medium *and higher than 500 as *high*. The *Specificity *field refers to the false positive rate associated to the motif (see Materials and Methods for details). A value of 10% indicates a *high *specificity, between 10% and 40% a *medium *specificity and above 40% a *low *specificity. For each motif a web-logo picture is shown, together with a schematic representations of the associated hydrogen bond patterns. For each motif, a structure multiple alignment of fragments containing the motif is also shown.

The A- [AS]- [FIV]- [NR]-A-P-L- [AT]-G motif is a pattern specific to voltage gated chloride channels (Pfam: PF00654). ClC channels are voltage-gated transmembrane proteins that catalyze the selective flow of Cl^- ^ions across cell membranes. From the structural point of view, the motif corresponds to two *reentrant *regions of ClC channels, *i.e*. protein regions that partially dip in the lipid membrane without crossing it entirely (see Fig. 1). No functional annotation about the protein regions carrying this motif is present in the Prosite database or in the Swiss-Prot Feature field. The structure of a ClC channel reveals two identical triangular subunits (gray and yellow in Fig. 1, PDB ID: 1kpl, chain B) related through a two-fold axis of symmetry perpendicular to the membrane plane and two parallel, independent pores. Each ClC Cl^- ^channel subunit contains 18 *α*-helices and exhibits a complex topology: the transmembrane *α*-helices within a subunit are tilted and variable in length and five of them have the typical features of reentrant regions. Three of the five reentrant regions are brought together near the membrane centre to form the selectivity filter for Cl^- ^ions, with their N-terminus dipoles pointing towards the binding site and creating a favourable electrostatic environment [27]. These regions are, for each subunit: GSGIP (106-110), GREWGP (146-150) and GIFAP (355-358), where residues *Ser*107, *Ile*356, *Phe*357, *Tyr*445 and *Glu*148 (PDB ID: 1kpl, chain:B) are annotated as chloride binding sites [27]. The A- [AS]- [FIV]- [NR]-A-P-L- [AT]-G motif is not part of the selectivity filter and no functional annotation for this motif is available. Nevertheless, this motif, found in *reentrant *regions at the interaction interface of the two ClC channel subunits forming the functional dimer, is highly conserved among bacterial voltage gated chloride channel sequences (data not shown). This evidence suggests that the motif must have a role in protein structural stability or dimerization and sheds light on novel functional aspects of voltage gated chloride channels. The large, stable interface between the subunits is expected because ClC Cl^- ^channels exist and function only as dimers [27]. Due to the electrical dipoles formed by the reentrant regions, the motif could contribute to provide a barrierless and energetically favourable environment for negatively charged particles present in the channel pore. In fact, it has been shown that, although the pore residues dominate the interaction with Cl^-^, other portions of the protein still contribute a significant fraction of the attractive interaction with Cl^- ^[28]. Detailed electrostatic calculations would be needed to assess if the reentrant regions at the dimer interface could contribute in a favourable way to the electric field around the Cl^- ^ions. The motif suggests that some residues are important for the protein function and could be tested experimentally for example by site-directed mutagenesis experiments and by checking if dimerization still takes place (e.g. through imaging with atomic Force Microscopy). The motif has been derived from the backbone torsion angle clustering of fragments of size 14 in the co-called *Reentrant *region (see Materials and Methods). It contains the conserved hydrogen bond pattern of regular *α*-helices between main chain atoms in relative positions 0 and 4, 1 and 5, 7 and 11, 8 and 12. The other residues in the fragment are not linked by hydrogen bonds between the backbone atoms, meaning that the regularity of the *α*-helix is broken to leave space for a small flexible loop characteristic of *reentrant *regions. The motif is highly specific to transmembrane proteins, with 23 occurrences in the Swiss-Prot-TM dataset (see Materials and Methods) and 0% false positive rate. The motif is specific to bacterial voltage-gated chloride channels and enriched in GO terms: *voltage-gated chloride activity*, *antiporter activity*, *chloride transport*, *chloride ion binding*. Furthermore, from the SCOPPI database [29] it has been found that some residues of this motif are positioned at the interaction interface between the ClC Cl^- ^channel subunits in the functional dimer (Fig. 1). Statistics and basic structural/functional annotation for this motif are shown in Fig. 2a.

#### [WY]-x(2)-Y-P-P-L: a membrane-water interface motif in heme-copper oxidases

The [WY]-x(2)-Y-P-P-L motif corresponds to a structured loop in the *Interface *region, close to the extracellular side of heme-copper oxidases (Pfam: PF00115). The heme-copper oxidases catalyse the reduction of molecular oxygen to water. The chemical energy released in the reduction reaction is utilized to transfer protons across the membrane and to generate an electrochemical proton gradient. They all have a low-spin heme, which is the initial electron acceptor, and a high-spin heme, which forms the catalytic site with a copper centre. The 6 histidine residues ligating the cofactors are fully conserved in the superfamily of heme-copper oxidases [30]. The heme-copper oxidase profiles are widely documented in the Prosite database (especially for cytochrome *c *oxidases). Also the His residues that ligate the heme groups and the copper ion are annotated in the Swiss-Prot Feature field. The pattern we find is not documented in any protein motif databases, but it is worthwhile to investigate its function as it contains a tryptophane residue (Trp164, PDB ID: 1ar1, chain A) which has been widely documented in literature. It has been shown that Trp164, which is hydrogen bonded to Δ-propionate of heme *a*_3 _in the catalytic centre, is highly conserved [30]. Mutation studies of this residue suggest that it is involved in regulating proton transfer from the pumping site near heme *a*_3 _to the P-side of the membrane [31].

**Figure 3:**
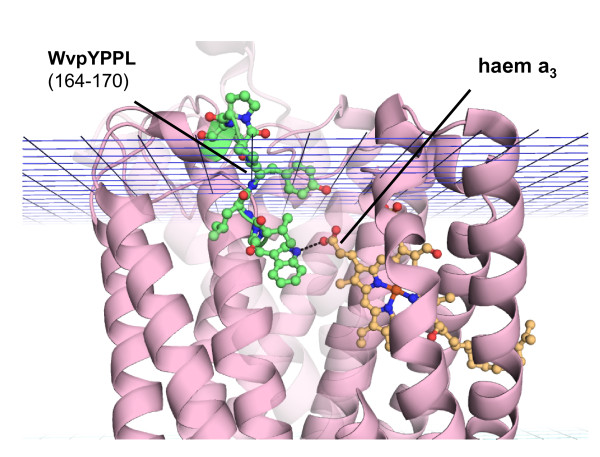
**[WY]-x(2)-Y-P-P-L motif**. PDB ID: 1ar1, chain A, cytochrome c oxidase. The structural motif corresponding to the [WY]-x(2)-Y-P-P-L sequence motif is highlighted using a ball and stick representation with carbon atoms in green, oxygen atoms in red and nitrogen atoms in blue. The hydrogen bond formed by Trp 164 with heme *a*_3 _is also shown as a dashed line.

We infer that the [WY]-x(2)-Y-P-P-L motif plays a special structural role in oxidases, creating the optimal environment to allow Trp164 (Tyr in cytochrome *ba*3 oxidases) perform its regulatory role in the proton transfer process. Furthermore, the motif suggests that Tyr164 plays the same role in cytochrome *ba*3 as Trp164 in cytochrome c. This evidence could guide future experiments in order to explore the detailed functional role of Tyr164, as, to our knowledge, no mutational studies of Tyr164 in cytochrome *ba*3 oxidases have yet been carried out. The motif has been derived from classes of fragments of size 7 and 8 clustered by backbone torsion angles. The structural motif associated to the sequence pattern contains the highly conserved residues Tyr167, Pro168, Pro169 and Leu179 (PDB ID: 1ar1, chain: A) which seem to form a highly structured cytoplasmic loop with the function of placing Trp164 at the right distance from and orientation with respect to heme *a*_3_. Our protein-ligand analysis also reveals Trp164 to be in close contact with the heme *a*_3 _group (Fig. 3). The [WY]-x(2)-Y-P-P-L motif has been found to be highly specific for transmembrane proteins, with 181 occurrences in the Swiss-Prot-TM dataset and enriched in the following GO terms: *oxidoreductase activity*, *heme binding*, *mitochondrial electron transport chain*, *iron ion binding*, *aerobic respiration*, *copper ion binding*, *electron transport*, *mitochondrion*, *metal ion binding*, *cytochrome-c oxidase activity*. Statistics and basic structural/functional annotation for this motif are shown in Fig. 2b.

#### L-x-S-I- [GP]: a convergently evolved interface helix motif

**Figure 4:**
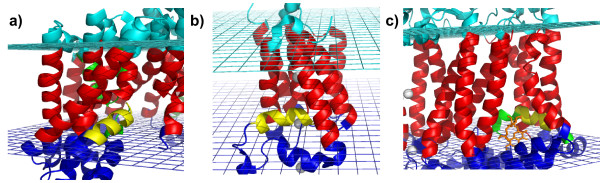
**L-x-S-I- [GP] motif**. a) Aspartate symporter (PDB ID: 2nwl, chain B). The L-x-S-I- [GP] motif is colored in yellow. The protein is colored according to the different regions with respect to the lipid bilayer: red *Helix core*, cyan *Cytoplasm*, blue *Extracellular *and green *Reentrant*. **b) Serine protease (PDB ID: **2ic8, **chain A). **The L-x-S-I- [GP] motif is colored in yellow. **c) cytochrome b (PDB ID: **1kb9, **chain C)**. The L-x-S-I- [GP] motif is colored in yellow.

The L-x-S-I- [GP] motif corresponds to a very short irregular helix almost parallel to the membrane plane (see Fig. 4) found across more than 80 protein families and derived from the structural clustering of fragments of length 10 in the *reentrant region*. This motif was found in proteins with different functions and it is a case of convergent evolution, *i.e*. proteins with different sequence and structure that share a common functional feature/mechanism. The structures from which the motif is derived are: aspartate symporter (PDB ID: 2nwl, chain: B), serine protease (PDB ID: 2ic8, chain: A) and cytochrome *b *(PDB ID: 1kb9, chain: C).

The aspartate symporter is a sodium-driven secondary transporter that catalyzes the uptake of aspartate from chemical synapses. It exists in the membrane as a trimer and each subunit has eight transmembrane segments, two reentrant helical hairpins (HP1 and HP2) and independent substrate translocation pathways [32]. It is thought that the HP2 reentrant region, where the L-x-S-I- [GP] motif is contained (see Fig. 4a), acts as a gate, adopting a open conformation and allowing the aspartate to reach the binding site from the extracellular solution [32]. The short parallel helix containing the L-x-S-I- [GP] motif is also involved in the formation of one of the two sodium binding sites (Ser349 and Ile350), as the transport of aspartate is highly coupled to sodium transport.

The serine protease, belonging to the Rhomboid proteases family, is a protein whose function is to cleave the transmembrane domains of other proteins. The crystal structure reveals six transmembrane segments and other two interesting features: an internal aqueous cavity that opens to the extracellular side and a long membrane-embedded loop between the first and the second helices [33]. The opening of this loop, which also contains the irregular helix corresponding to the L-x-S-I- [GP] motif (residues 137-145, PDB ID: 2ic8, chain A, see Fig. 4b), is thought to be the likely route by which the substrate enters the active site. So, it has been postulated that this loop, and in particular the segment corresponding to the motif, functions as a gate and may change conformation when the substrate binds [33]. Finally, there is no evidence in literature that the same motif in cytochrome *b *(see Fig. 4c) is associated to a gating function. But it has been found, from ligand analysis, that the structural segment corresponding to the L-x-S-I- [GP] motif is part of the *Q*_0 _binding site and involved in non-covalent interactions with the substrate. This suggests that the motif (residues 278-287, PDB ID: 1kb9, chain C) is involved in conformational changes upon substrate binding, but this assumption needs experimental validation. It can be concluded that these three proteins, unrelated in structure and biochemical function, share a convergently functional motif that, although not directly part of the core catalytic activity of the protein, modulates gate dynamics at the membrane-protein interface. Statistics and basic structural/functional annotation for this motif are shown in Fig. 2c.

### General results from the structure fragment clustering

**Figure 5:**
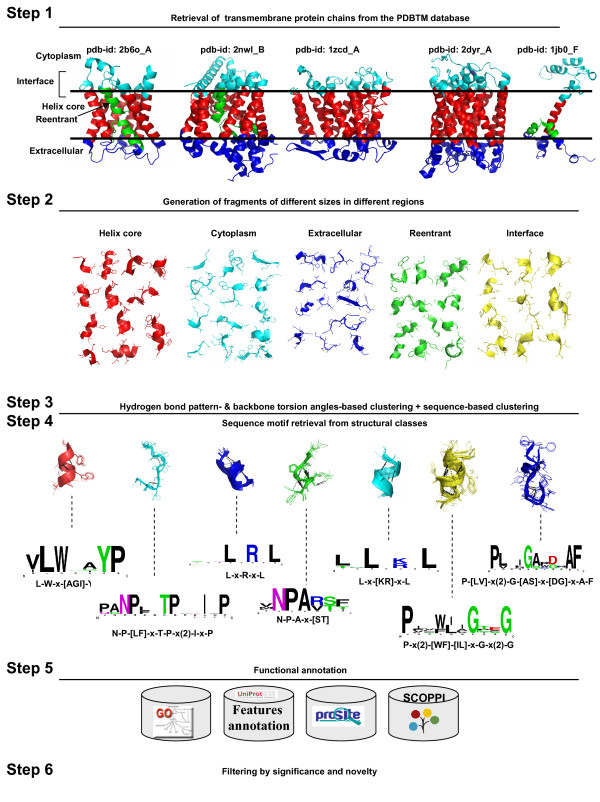
**Workflow of the method**. Workflow of the method. **Step 1 **Retrieval of 168 transmembrane protein chains from the PDBTM database. Some of them (PDB IDs: 2b6o_A, 2nwl_B, 1zcd_A, 2dyr_A, 1jb0_F) are shown. For each chain, the PDBTM annotation for the location of the chain with respect to the lipid bilayer planes is shown. The exact definition of the different regions is given in Materials and Methods. **Step 2 **Fragments of sizes in the range from 3 to 14 amino acids are generated from the protein chains and classified according to the region they belong to. **Step 3 **For each size and region fragments are clustered according to similar hydrogen bond patterns, backbone torsion angle profiles and sequence similarity. **Step 4 **Sequence motifs are generated for each structural class. **Step 5 **Functional annotation is generated by means of GO, Swiss-Prot, Prosite, and SCOPPI databases. **Step 6 **Sequence motifs are filtered according to their statistical significance, their specificity for transmembrane proteins, and their novelty.

Consider Fig. 5, which describes the procedure of structural fragment clustering. It consists of six steps. In step 1 non-redundant sequences (NR 90%) and transmembrane protein structures with resolution less than 3.5 Å are collected. In step 2, the protein structures are fragmented and fragments are labeled according to their location and topology. In step 3, the fragments are clustered based on their hydrogen bonding patterns and on torsion angles, respectively. In step 4, sequence motifs are derived from significant clusters of fragments and in step 5, they are annotated regarding functional and structural features. In the final step 6, all motifs are filtered regarding their significance and novelty.

#### Structural fragment clustering

In step 1 non-redundant sequences (NR 90%) and (transmembrane protein structures with resolution less than 3.5 Å were collected.

In step 2 fragments of different lengths, ranging from 3 to 14 amino acids, were generated from a set of 168 non-redundant *α*-helical membrane protein chains from the PDBTM database [34]. Structural fragments were assigned to different regions with respect to the position of the lipid bilayer, based on the PDBTM annotation. For each fragment, a backbone torsion angle profile was derived from the corresponding PDB file, and the associated hydrogen bond pattern, when one existed, was calculated by means of the Chimera algorithm [35].

In step 3, hierarchical clustering of fragments of the same length and region was performed by implementing two different distance measures: one based on similar backbone torsion angle profiles and the other one based on similar hydrogen bond patterns. The latter is a novel distance measure that we defined and, to our knowledge, it was never used before in other fragment clustering approaches. The reason for this is the that 3D/sequence motifs can be region-specific and show specific hydrogen bond patterns (e.g. Schellmann motifs, binding sites) but unspecific backbone torsion angle profiles. Motifs can also lack a specific hydrogen bond pattern but be detectable by means of their Φ and Ψ angle values (kinks in transmembrane helices, reentrant regions). A similarity measure based on common hydrogen bond profiles can reveal very stable structural motifs, as hydrogen bonds in membrane proteins are much stronger than in globular proteins (even when the donor-acceptor distance is around 4 Å). This is due to the low dielectric constant of the membrane and lack of competing interactions with water molecules [36]. On the other hand, it has been shown that a similarity measure based on differences in backbone torsion angle profiles is very sensitive to variations in local protein conformations and that active site torsion angles are usually highly conserved [37].

In step 4 sequence motifs (regular expressions) were derived for each structural class, if possible. If no significant motif could be associated with a cluster, a further sequence-based clustering step was performed to filter significant sequence patterns. Then, a library of regular expressions associated with specific structural features was compiled. In step 5, functional annotation of fragments was performed using multiple sources of information. Fragment clusters were annotated with shared GO categories [38]. Each fragment in a cluster was associated to a Swiss-Prot feature FT field annotation, when this annotation existed. Fragments that belong to protein-protein interfaces or are part of ligand/substrate binding site were annotated by mapping them onto the SCOPPI [29] and the PDB database [39] for possible binding sites. Furthermore, residues in fragments that have been experimentally mutated and have a function reported in the literature were also annotated. Finally, for each cluster-derived sequence pattern, its total or partial overlap with a PROSITE [40,41] pattern is checked and reported.

In step 6, the final step, the motifs were filtered. Initially, there were 4842 motifs. First, we filtered by significance and specificity to membrane proteins, resulting in 2228 motifs. Next, we filtered out motifs that are already documented in general motif databases, resulting in 213 novel motifs. Finally, we grouped overlapping motifs and thus remove redundancy, leaving 58 motifs. These 58 motifs are described in detail regarding structural and functional features in Additional file 1.

#### Statistics of clusters and filtering

The total number of clustered fragments was 215.518 for the hydrogen bond-based clustering and 370.546 for the backbone angle-based one (not all fragments have an associated hydrogen bond pattern). In Table 1, the statistics for both clusterings are summarized: number of fragments, structural clusters, outliers and size of the largest cluster for each region and fragment length. Plots of these numbers versus fragment length are presented and discussed in Additional file 2. The number of clusters and outliers (clusters containing a single element) is low in the *Helix core *region with respect to the *Cytoplasmic*, *Extracellular *and *Interface *regions. This is due to the low structural diversity of the *Helix core *region, where 80% of the fragments fall into the regular *α*-helix structural class or irregular 3_10 _helices (see Additional file 3). In the *Cytoplasmic *and *Extracellular *regions, the number of classes increases and their distribution drastically changes since there is more structural variability compared to the *Helix core *region.

**Table 1:** Overall statistics for the generated clusters.

	Hydrogen bonding clustering				Torsion angle clustering			
	**Region Cytoplasm**				**Region Cytoplasm**			
**length** **in aa**	**fragments**	**clusters**	**outliers**	**largest** **cluster**	**fragments**	**clusters**	**outliers**	**largest** **cluster**

3	641	13	0	166	9669	11	0	294
4	2307	25	0	1182	9281	28	2	1722
5	4911	46	0	1901	8908	52	4	4656
6	5602	63	0	1796	8550	74	7	3656
7	6001	95	2	1804	8214	100	7	4621
8	6213	99	4	2185	7896	78	8	4810
9	6330	144	12	1997	7593	74	13	5837
10	6366	171	19	2038	7308	78	22	5894
11	6347	165	25	2428	7039	71	21	5899
12	6282	147	27	2822	6785	64	26	5943
13	6188	157	37	3837	6546	73	28	5843
14	6075	132	36	3745	6319	78	29	5688

	**Region Extracellular**				**Region Extracellular**			

3	621	13	0	169	9236	20	0	198
4	2234	26	0	1086	8848	34	2	1435
5	4865	45	0	1837	8477	42	6	4168
6	5557	69	0	1750	8126	66	17	3690
7	5941	97	2	2048	7797	72	17	4445
8	6125	123	10	2094	7481	77	19	5587
9	6179	139	19	2108	7176	74	15	5671
10	6160	167	27	2337	6886	70	14	5846
11	6089	152	32	1441	6613	84	15	5655
12	5989	160	40	2617	6359	71	8	5650
13	5863	167	44	2549	6118	81	8	4404
14	5717	156	36	2678	5895	78	11	5361

	**Region Helix core**				**Region Helix core**			

3	83	12	2	14	10753	22	4	27
4	1111	23	3	48	10131	27	11	1020
5	8327	32	8	7751	9509	33	12	8340
6	8335	36	16	7247	8887	36	12	8374
7	7912	36	18	7256	8265	30	10	8054
8	7357	27	9	6978	7643	32	14	7491
9	6771	24	6	6722	7021	43	14	6864
10	6178	23	6	6243	6399	43	17	6253
11	5581	17	4	5687	5777	41	17	5648
12	4981	13	2	5116	5158	39	18	5064
13	4382	20	10	4503	4541	42	22	4437
14	3785	17	9	3900	3925	42	21	3837

	**Region Interface**				**Region Interface**			

3	114	13	2	23	2433	16	3	52
4	682	25	3	430	3608	27	5	385
5	3715	40	4	2228	4749	40	9	3214
6	5122	56	8	3503	5847	64	11	3964
7	6352	71	11	4576	6885	68	17	5071
8	7480	74	8	5770	7881	77	23	6346
9	8533	82	15	6952	8842	82	29	7311
10	9522	83	19	8200	9758	100	26	8860
11	10429	86	19	9202	10621	122	27	9285
12	11263	84	25	10123	11422	149	34	9687
13	12039	85	27	11142	12175	168	29	10400
14	12766	79	25	1207	12883	180	33	9907

	**Region Reentrant**				**Region Reentrant**			

3	21	7	1	6	313	4	2	9
4	65	12	1	32	291	10	3	35
5	204	14	3	135	269	11	6	151
6	210	19	5	123	247	10	8	143
7	205	20	6	122	225	15	9	128
8	193	16	4	128	203	15	10	123
9	177	12	4	126	181	14	10	116
10	158	10	4	134	159	15	9	91
11	137	14	9	112 20	137	18	10	55
12	116	14	7	57	116	18	8	36
13	95	11	6	45	95	19	5	21
14	77	19	10	26	77	20	10	14

Table 2 shows the number of structural classes that could be assigned to a sequence pattern for both hydrogen bond and torsion angle clustering. The first column shows the number of classes obtained for the hydrogen bond clustering before and after the sequence-clustering step. The second column shows the percentage of clusters for which a sequence pattern could be derived before and after the sequence-clustering step. The third column shows the percentage of classes that could be assigned to a statistically significant motif before and after the sequence-clustering step. Columns four, five and six show the same numbers for the torsion angle clustering. Note, only 0.4% of the structural classes (for the hydrogen bond clustering) share some signal at sequence level, compared to the 3.6% of the torsion angle clustering. After the sequence clustering step the number of statistically significant motifs drastically increased to 17% for the torsion angle clustering and 30% for the hydrogen bond clustering. This evidence suggests that the backbone torsion angle-based distance measure is a better approach for direct sequence-structure correlations. On the other hand, some specific structural motifs, associated with a significant pattern, could be detected only after hydrogen bond clustering.

**Table 2:** Clusters and sequence motifs.

Structure-based clustering only					
**Hydrogen bonding clustering**			**Torsion angle clustering**		

**clusters**	**% cov by motif**	**% cov by significant motif**	**clusters**	**% cov by motif**	**% cov by significant motif**
2597	6	0.4	1747	9.7	3.6

**Structure-based and sequence-based clustering**					

**Hydrogen bonding clustering**			**Torsion angle clustering**		

**clusters**	**% cov by motif**	**% cov by significant motif**	**clusters**	**% cov by motif**	**% cov by significant motif**
7170	54.2	30.0	8866	39.1	17.1

Percentage of structural classes covered by sequence patterns. The first column shows the number of classes obtained for the hydrogen bond-based clustering before and after the sequence-clustering step. The second column shows the percentage of clusters for which a sequence pattern could be derived by using the Pratt program before and after the sequence-clustering step. The third column shows the percentage of classes that could be assigned to a statistically significant motif before and after the sequence-clustering step. Colums four, five and six show the same numbers for the backbone torsion angle-based clustering.

In total, 4843 non-redundant sequence motifs have been derived from both clusterings. From this number, the statistically insignificant motifs and those motifs that are not specific to transmembrane proteins were filtered out. A motif is considered statistically significant when its associated p-value, derived by randomly permutating the Swiss-Prot TM database, is smaller than 0.05 (see Material and Methods). Furthermore, a motif is considered specific to transmembrane proteins if its false positive rate is low enough, *i.e *if the p-value of the hyper-geometric distribution is smaller than 2 × 10^-6 ^(see Material and Methods).

**Figure 6:**
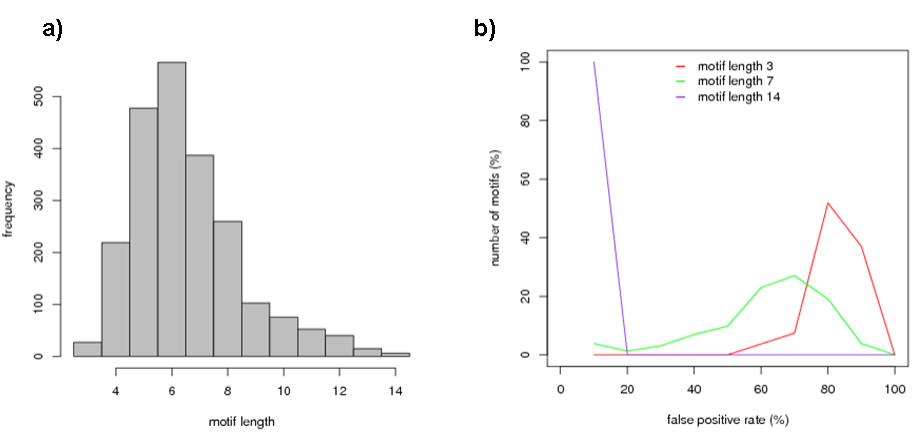
**Distributions**. a) Distribution of the derived statistically significant motifs for different motif lengths. **b) **Percentage of motifs vs false positive rate for motifs of lengths 3, 7 and 14.

Fig. 6a shows the distribution of motifs of different lengths. The histogram shows that the strongest correlation between structural clusters and sequence preferences is obtained for motifs of length of 5 to 7 amino acids. The number of sequence patterns associated to structural classes strongly decreases for fragment length greater than 8 amino acids. Fig. 6b shows the percentage of motifs associated with different false positive rates for three different fragment lengths (3, 7 and 14 amino acids). An anti-correlation has been observed between false positive rate and motif length (Pearson correlation -0.76): the longer the sequence motif, the lower the false positive rate. As shown in Fig. 6a, for length 14, 100% of the motifs have false positive rate less than 20%; motifs of length 3 are very unspecific as most of them have false positive rate greater than 60%.

After the filtering step, 2228 significant motifs were considered for further analysis. The average resolution of the structural motifs after filtering is 2.45 Å. This means that the clustering process automatically filtered out lower resolution fragments as outliers. 85% of the derived motifs were common to both hydrogen bond- and torsion angle-based clusters (data not shown), especially in the *Helix Core *region. This observation is a further proof of the reliability of the retrieved structural motifs. In order to evaluate the ability of our structure-based method in finding novel motifs, which cannot be identified by sequence-based methods alone, an all-against-all comparison of our motifs against the Prosite database was carried out. The algorithm used for comparison with Prosite patterns is described in detail in Materials and Methods.

It is necessary to stress that a direct comparison of our motif library with the Prosite database is not straightforward for three main reasons. First, as Prosite patterns are derived at sequence level, from conserved regions in multiple alignments of homologous sequences, they are usually longer (15 to 20 amino acids on average) than ours (3 to 14 amino acids). Second, Prosite patterns are derived by scanning all the sequences in the Swiss-Prot database, unlike our library that is derived only from transmembrane proteins of known structure. For this reason the comparison is limited to those Prosite patterns that hit anywhere in Swiss-Prot transmembrane proteins (Swiss-Prot-TM dataset). Third, it has to be taken into account that the number of known structures for membrane proteins is considerably smaller than the number of known sequences. This implies that a high coverage value of our motif library against Prosite cannot be expected. On the other hand, it is interesting to quantify and investigate the number of found linear motifs that do not have any match in Prosite, as they provide a proof that structural information adds new knowledge about unannotated sequences and functional implication. By analyzing the results from the comparison, it has been found that by varying the value of the cut-off for defining a match between two patterns, the number of matched/unmatched motifs strongly varies (see Additional file 4). The cut-off for the similarity score was set to 0.86. The choice of the cut-off is based on a comparison to a random model (see Additional file 4). By setting the cut-off for the similarity score to 0.86, the percentage of significant novel motifs is about 10%, 213 motifs out of 2228 non-redundant, statistically significant motifs. Although a comparison with Prosite is not straightforward, it is clear that the 213 novel motifs represent new knowledge, which cannot be gained from sequence alone.

After grouping overlapping motifs and removing redundancy, 58 motifs were checked in the scientific literature for the first biological assessment and described in detail regarding structural and functional features, in Additional file 1.

### Comparison with MEME

In order to evaluate the capability of our structure-based method to survey motifs that cannot be found by sequence-based pattern searching tools alone, we compared our motifs with the motifs obtained by using the MEME tool, a software package to discover motifs in groups of related DNA or protein sequences [42]. We derived motifs with MEME on the same dataset of 168 protein chain sequences used for generating our motifs. In total, 98 motifs were generated using MEME, with the following options: motifs lenght ranging from 3 to 14 amino acids; motifs generated from a minimum of 5 sequences; e-value less that 10.0.

In oder to compare our motifs with MEME-generated motifs, we computeted the overlap between couples of motifs by counting the number of common transmembrane proteins in Swiss-Prot where the two motifs hit. If the overlap was higher than 80% the motifs were considered as the same motif. We find that less than 20% of our motifs could be found by MEME. In particular, the three motifs corresponding to the three examples discussed in the previous sub-section could not be detected from the MEME tool. Out of the 58 motifs described in Additional file 1, 15 could also be found by MEME and they all correspond to family-specific motifs. 43 motifs out of 58 could only be found by means of our structure fragment clustering approach and represent new knowledge that cannot be derived by means of sequence-based pattern searching tools alone. Since a key finding of this paper is that motifs exist as modular building blocks across unrelated families, it is clear that purely sequence-based approached are not adequate due to the divergence of the different families.

### Family vs non-family specific motifs

**Figure 7:**
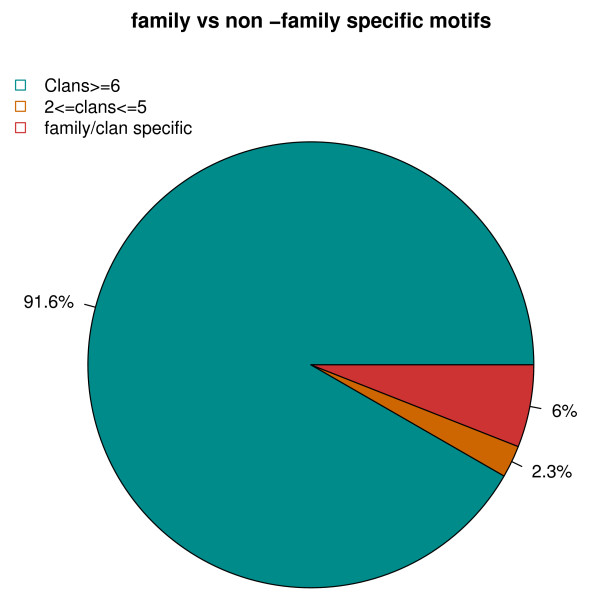
**Motifs and Pfam families**. Percentage of family-specific motifs versus cross-family motifs. About 91% of all motifs have been found in more than 6 different Pfam clans. Only 2.3% of them are found in less than five Pfam clans. The family specific motifs cover only 6% of our motif library.

The sequence homology of the dataset for deriving the motifs is reduced at 90% identity. This is a quite permissive cut-off and the risk of deriving only homology-based patterns, representative of a limited sampling, is high. To verify that not all the patterns we derive are obvious signals of homologous proteins, but that many of them are functionally or structurally of great importance (even if not homology-derived), an analysis at family level was carried out by using the Pfam database. Each motif can be assigned to a Pfam family or to a set of Pfam families by assigning a Pfam family to the Swiss-Prot-TM sequences the motif matches. Surprisingly, we found that only 6% of the 213 statistically significant motifs are family-specific. The rest of the motifs are found across different Pfam families or clans (see Fig. 7). Novel family-specific motifs, not represented in the Prosite database, are interesting because they can shed light on novel and different aspects of a protein's structure and function. Motifs across families can be important for structural stability, *e.g*. transmembrane helix kinks, motifs at the membrane-water interface, protein-lipid interaction motifs, helix-helix packing motifs, or they can be cases of convergent evolution, *i.e*. found in proteins that are not homology-related but share some functional mechanisms. The A- [AS]- [FIV]- [NR]-A-P-L- [AT]-G and [WY]-x(2)-Y-P-P-L motifs discussed in the previous sections are examples of motifs specific to the voltage-gated chloride channel family and the heme copper oxidase family, respectively. The L-x-S-I- [GP] parallel helix motif, also discussed in the previous section, is an example of motif found across different protein families and it is a potential convergent evolution motif.

Other important motifs across families, such as helix kinks, helix distortions, interface helices, helix-helix packing and protein-lipid interaction motifs are described in Additional file 1.

### Motifs help to identify membrane proteins in novel genomes

**Figure 8:**
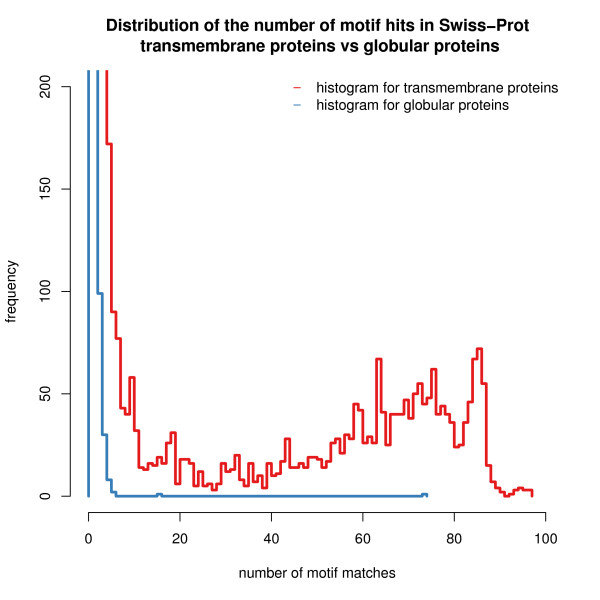
**Motif hits in Swiss-Prot transmembrane proteins vs. globular proteins**. Distribution of the number of high-confidence motif hits in transmembrane proteins (red histogram) vs. globular proteins (blue histogram) in the Swiss-Prot database. The plot shows that almost all globular proteins do not exhibit more than 5 different motif hits per sequence, unlike transmembrane proteins, which contain more than five motif hits (and up to 100 matches). This allows to conclude that it can be possible to identify and characterize a new protein sequnce as 'transmembrane' if the number of different motifs hits from our library is higher than five.

To demonstrate the capability of our motifs to identify new transmembrane proteins, we performed the following analysis: we determined the distribution of the number of motif hits in Swiss-Prot sequences for transmembrane proteins against globular proteins. The two distributions are shown in Fig. 8. This analysis was performed for high-confidence motifs, *i.e*. motifs with false positive rate less than 40% as defined in Additional file 1. Fig. 8 shows that all globular proteins (blue histogram) contain five or less motif hits, in contrast to transmembrane proteins which can contain up to 100 motif hits. This allows to define a simple rule: if a new sequence contains more than 5 of the motifs, then it is predicted to be a transmembrane protein. Applied to Swiss-Prot this rule does not make any false predictions. However, since the motifs are based on structures, the coverage is not high and thus there will be still many membrane proteins among the sequences for which no prediction is made. In this respect, our method can help identifying transmembrane proteins in novel genomes.

### Coverage of motifs in different genomes

The coverage of the 58 non-redundant motifs in different genomes has been estimated and the results presented in Table 3. Table 3 shows the percentage of transmembrane proteins in the Swiss-Prot database, where at least one motif hits, for the top ten genomes, *i.e*. the genomes with the highest number of motifs hits, ordered by the decreasing number of the motif hits. In average, the coverage of the portion of genome encoding for membrane proteins is 9%. 293 transmembrane proteins were found to be associated with at least one of the 58 motifs in the human genome.

**Table 3:** Motifs across species.

Species	Number of motif hits	Number of Swiss-Prot proteins	Percentage
Homo sapiens	293	4647	6.0
Mus Musculus	249	3723	7.0
Escherichia coli	163	1911	9.0
Rattus norvegicus	156	1951	8.0
Saccharomyces cerevisiae	103	1414	7.0
Arabidopsis thalia	100	1252	8.0
Drosophila melanogaster	73	624	12.0
Staphylococcus aureus	70	1076	7.0
Bos taurus	70	1019	7.0
Salmonella	63	713	9.0

Percentage of transmembrane proteins, containing at least one of the 58 non-redundnat, fully characterized sequence motifs, for each species. Only the top 10 mostly represented species, oredered by the number of motif hits in the dataset of transmembrane proteins are reported. The first colomn specifies the protein species, the second colomn specifies, for each species, the number of motif hits in Swiss-Prot transmembrane sequences, the third colomn specifies, for each species, the number of corresponding transmembrane protiens in Swiss-Prot and the last colomn shows, for each species, the percentage of transmembrane protein that contains at least one motif.

More generally, we estimated also the coverage of the 58 motifs in three protein kingdoms. It was found that 15% of eukaryotic proteins were covered by the 58 motifs, against 9% in Bacteria and Archaea.

## Conclusions

In this work we introduced structural fragment clustering and derived 213 novel sequence motifs in membrane proteins together with their functional characterization The novel motifs appear across many families and therefore show that they form functional modules, which are re-used. The majority of the motifs is found on binding sites to membrane, ligands, co-factors, other proteins or other helices, highlighting their functional role and the importance of the environment for these structural building blocks (see Additional File 1).

We discuss three novel motifs in detail. Two of them, a re-entrant region in voltage-gated chloride channels and a structured loop in the membrane-water interface region of heme-copper oxidases, are family-specific and help add precious details to the functional mechanisms of the proteins they are found in. The third motif, an interface helix derived from the clustering of fragments in three different protein families, is an interesting case of convergent evolution, where three evolutionarily unrelated families with different functions share a common gating mechanism. The three motifs discussed here have been chosen among 213 novel statistically significant and non-redundant motifs derived by means of an unsupervised learning method also described here.

The method uses structural information about protein fragments, like conserved hydrogen bond patterns and backbone torsion angle profiles, to derive short linear motifs in *α*-helical transmembrane proteins. Although the data set contains low-resolution structures with a resolution worse than 2.5 Å, 98% of the clusters contain at least one high-resolution fragment from a structure of less than 2.5 Å resolution. This garantees reliability of the results. Furthermore, the distribution of the average resolution value for all clusters has a peak around 2.5 Å (see Additional file 5). Due to intrinsic difficulties in experimental determination of membrane protein structures [43], their average resolution in the PDB is worse than for globular proteins (2.9 Å vs.2.18 Å) [44]. Removing structures of resolution worse than 2.5 Å and filtering for redundancy would reduce our dataset from 97 to only 40 structures. This would make it nearly impossible to perform clustering and to obtain statistically significant results.

The method, even though it is based on the information retrieved from the limited set of membrane proteins with known 3D structures, is able to find novel functionally or structurally important motifs that can complement and enrich information retrieved from sequence-based methods like Prosite or MEME. While new protein domains or family signatures, such as those contained in Pfam [45] or Prosite [41], can be defined from alignments of evolutionarily related sequences, the identification of short sequence motifs, related to specific structural features and shared between transmembrane protein functional classes, is much harder. To address this problem different sources of information are taken into account to elucidate the role played by short structural/sequential motifs in different transmembrane proteins classes. These are: structural properties, location with respect to the membrane planes, GO annotations, Swiss-Prot functional annotations, interaction interface information, and mutational analysis. It is shown in the three examples that the method is able to predict new functional residues. In future studies, the method will be applied to better characterize other important sequential/structural motifs in transmembrane proteins, like interfacial helices or patterns at helix-helix interaction interfaces.

Our method, in contrast to the approach described in [26], not only enables the discovery of 3D motifs associated with function, but makes use of regular expressions to allow searching for functional motifs in transmembrane proteins of unknown structure. Cluster signatures are an attractive way to annotate protein function at both the structure and the sequence levels. Indeed, it has been shown that protein annotation effort benefits immensely from the knowledge of functional signatures in both primary, secondary and tertiary structure. In fact, sometimes the multifunctionality and overall structural diversity of even closely related proteins confounds efforts to assign function on the basis of overall sequence or structural similarity [46]. Approaches based on the identification of common small functional motifs can help to overcome this problem. This is especially true for membrane proteins, where protein with the same topology, fold or signal at sequence level in the hydrophobic core, perform totally different functions, thanks to hotspot residues and small motifs that differentiate them, or share common functional mechanisms (e.g. see convergently evolved motif L-x-S-I- [GP] in the Results section).

In addition, functional motifs pinpoint individual residues that play a crucial functional role and complement the information contained in alignments of homologous proteins, such as the ones contained in Pfam, which focus on large functional signatures, at family level, but not on individual residues or small motifs, related to specific structural features.

Our method generates structural motifs and associate them directly to function by mapping onto the protein fragments functional characteristics, such as Swiss-Prot 'features', protein-protein interface and protein-ligand binding sites associated residues and Go annotation. Furthermore, fragments belonging to functional sites or containing hot-spot residues are ideal candidates for experimental validation. Possible experiments that can be done to validate the structural and functional role of a motif involve different experimental techniques. First, force spectroscopy, which allows to measure the force necessary to pull proteins out of the native membrane, can be used to validate structural motifs important for protein stability [47]. Second, confocal microscopy can validate motifs relating to the protein's topology, such as interface helices, by labeling membrane and motif with different dyes and by determining the location of the motif seuence relative to the membrane. Third, mutants from membrane proteins can be tested by means of functional assays. For example, for the dimerization motif in chloride channels, dimerization upon mutation of one of the conserved residues in the motif can be tested by means of imaging through atomic force microscopy (looking at the protein in its native environment). If dimerization still takes place, it can be tested whether the dimer is a functional dimer, e.g. by measuring the concentration of Cl^- ^and H^+ ^exchanged. To conclude, it is worthwhile to emphasize the power of the method and the results presented here in two other application fields. First, motifs from our library can help identifying transmembrane proteins in novel genomes, as discussed in Results and Discussion. Second, structural motifs, such as reentrant regions, helix kinks and helix-helix contact motifs or functional motifs, such as the ones related to the protein binding sites or protein-lipid interactions, can be used as constraints while building more refined two-dimensional models of *α*-helical transmembrane proteins from sequence alone. It has been shown that both two-dimensional tools [22] and three-dimensional prediction algorithms benefit from the use of structure-sequence motifs as constraints.

## Methods

### Dataset

The source of transmembrane protein sequences for this work was the PDBTM database, a comprehensive and up-to-date selection of transmembrane proteins from the Protein Data Bank (PDB) [34]. The database contains 792 transmembrane structures (as of August 17, 2007), 671 of which are alpha-helical membrane proteins. The redundant number of alpha-helical membrane protein chains that contain at least one transmembrane segment is 2135. From this list files corresponding to theoretical models, cryo-electron microscopy structures and X-ray structures solved at worse than 3.5 Å resolution are eliminated from the dataset, as they are considered of low resolution. From the filtered set, a list of non-redundant transmembrane protein chains is selected by reducing the sequence identity between them with CD-HIT [48]. Redundant sequences at 90% sequence identity are removed and the structures with highest resolution are chosen as representatives of each CD-HIT output classes. At the end, our filtered dataset contains 168 non-redundant *α*-helical transmembrane protein chains from 97 different PDB structures, whose average resolution is 2.54 Å.

### Fragments generation and description

Fragments of different sizes are generated using a sliding window of length ranging from 3 to 14 amino acids.

#### Structural description

##### Hydrogen bond patterns

A set of hydrogen bonds between side-chain and/or main-chain atoms of its residues is assigned to each fragment. For example, if a given fragment has the following pattern ((*N*,0, M, *OE*1,1, S),(*NZ*,2, S, *O*,4, M)), this means that the fragment contains two hydrogen bonds: one between the main-chain (indicated with M) nitrogen atom at relative position 0 with the side-chain (indicated with S) oxygen *OE*1 at relative position 1 and the other one between the side-chain atom *NZ *at relative position 2 and the main-chain oxygen at relative position 4. Hydrogen bonds are detected by means of the Chimera algorithm *FindHBond*, which uses atom type and geometric criteria to identify putative hydrogen bonds [35,49].

##### Backbone torsion angles

Each fragment is associated with the list of backbone torsion angle values (Φ and Ψ) of its residues, taken from the PDB file of the protein chain the fragment belongs to.

##### Location with respect to the membrane

Each fragment is assigned to a given region with respect to the lipid bilayer planes through a slightly modified version of the PDBTM annotation. The PDBTM database contains for each molecule the most likely localization of the membrane relative to the molecule, and each CHAIN record contains one or more REGION records that locate the chain segment in the space relative to the membrane [34,50]. The region types each fragment can be assigned to are: *Cytoplasmic*, *Extracellular*, *Helix core*, *Reentrant *and *Interface*. *Cytoplasmic *and *Extracellular *refer to the two sides of the membrane, *Helix core *to the inner membrane part of *α*-helical membrane proteins, *Reentrant *to membrane-loop structures that correspond to polypeptide chains that do not cross the membrane but just dip into it (like in aquaporin or potassium channels), and *Interface *to membrane-water interface regions. The *Interface *region is a modification we introduce, with respect to the PDBTM annotation, for fragments that cannot be unequivocally associated with a region but comprise part of the *Helix core *and *Cytoplasmic *or *Extracellular *region. As the annotation regarding *Cytoplasmic *and *Extracellular *region in the PDBTM database is not explicitly stated, and instead the two sides of the membrane are called *Side 1 *and *Side 2*, the assignment was based on the topology annotation contained in the TOPDB database [51]

### Generation of the motif library

#### Clustering

##### Hydrogen bond-based clustering

The distance measure is proportional to the absolute number of common hydrogen bonds between two fragments, where two fragments are said to share the same hydrogen bond if the residues involved in the bond occupy the same relative positions inside the fragments and have the same atom type involved in it. The distance *d*_*HB *_is the following:(1)

where the second term is the similarity score between two fragments, corresponding to the number of common hydrogen bonds between fragments *f*_1 _and *f*_2_:(2)

The functions *hb*(*f*_1_) and *hb*(*f*_2_) are the number of hydrogen bonds in fragment *f*_1 _and *f*_2_, respectively. Fragments *f*_1 _and *f*_2 _can share three different types of hydrogen bonds: main chain-main chain, *MM*, side chain-main chain, *SM *(and vice versa), and side chain-side chain, *SS*. For this reason *sim*_*HB*_(*f*_1_, *f*_2_) can be expressed as the sum of three terms:(3)

where *w*_*MM*_, *w*_*SM *_and *w*_*SS *_are weights given to the three different hydrogen bond types and correspond to the inverse number of occurrences of main chain-main-chain, side chain-main chain and side chain-side chain hydrogen bonds, respectively. In this case the values of *w*_*MM*_, *w*_*MS *_and *w*_*SS *_are 0.0055, 0.025 and 0.12. The similarity score between two fragments, *sim*_*HB*_, is normalized by means of min-max normalization. Given the following scores {*s*_*k*_}, *k *= 1,... *n*, the normalized scores are .

##### Backbone torsion angle-based clustering

The distance measure is proportional to the difference in Φ and Ψ torsion angles over the two fragment residues. Let the two *n*-length fragments have sequences of torsion angles (Φ_1_, Ψ_1_),...,(Φ_*n*_, Ψ_*n*_) and . Let ΔΦ_*i *_be the difference of the corresponding Φ angles of the two fragments and ΔΨ_*i *_be the difference of the corresponding Ψ angles. We define the distance *d*_*T *_as:(4)

The distance measure between two fragments is normalized by means of min-max normalization. For each sub-cellular region and fragment size, fragments are clustered by means of hierarchical clustering with average linkage. The cluster is cut, and structural classes are generated according to a criterion that maximizes the number of correctly positioned fragments inside a sub-cluster and minimizes the RMSD value of the structural alignment of fragments inside the sub-cluster.

##### Sequence-based clustering

After deriving structural classes, a further filtering at the sequence level might be needed for deriving specific sequence patterns. The distance measure used for the sequence-based clustering procedure is proportional to the sum of the BLOSUM50 substitution scores between corresponding residues. The normalized distance *d*_*S *_between two fragments *f*_1 _and *f*_2 _is:(5)

where *n *is number of residues in the fragment and *blosum*50(*f*_1 _[*k*], *f*_2 _[*k*]) is the substitution score between the corresponding amino acids at position *k *in the fragments. The sequence-based tree is cut according to an empirical criterion that minimizes the number of outliers (sub-clusters with only one element) and the number of clusters with fewer than 5 objects.

##### Sequence patterns

Sequence motifs, in the form of regular expressions, are generated for each sub-cluster by means of the program Pratt [52], an algorithm that, given a set of unaligned protein sequences (fragment sequences in our case), finds patterns matching a given number of these sequences. The program uses Prosite notation to describe the patterns. For this special application, we choose a value of 80% for the minimum percentage of sequences to be matched inside a sub-cluster when sequence motifs are generated directly after the structure-based clustering and a value of 100% when motifs are generated after the sequence-based clustering.

### Prosite comparison by aligning regular expressions

For the comparison of our motif library with known motifs in the Prosite database we first filter the Prosite patterns that are found to occur in the Swiss-Prot dataset of transmembrane proteins (Swiss-Prot-TM). This number is equal to 456, about 35% of the total number of Prosite patterns. In order to determine which of our motifs can be considered novel, all regular expressions from our motifs are directly compared against the 456 Prosite patterns. We check if a motif is contained within, or overlaps over a given threshold with a Prosite pattern, by progressively sliding the patterns on top of each other. Then every possible 'fit' between two patterns is scored according to a well defined scoring scheme and matches between a motif and a Prosite pattern (or a portion of it) are defuned when the similarity score between the two stretches compared exceeds a reasonable cut-off. In detail, when comparing two patterns or two regular expressions, segments of the same length are compared. The similarity score between two segments of the same length is defined as follows:(6)

where *l *is the segment length (in terms of symbol positions) and *pair_score *is the score between two corresponding symbols in the two regular expressions. The score between two symbols is calculated in the following way. It is assumed that *M*_1 _is a motif from our library, *M*_2 _is a portion of a Prosite pattern and *i *= 1,...., *l *is the position of a symbol in both *M*_1 _and *M*_2_. A symbol can be the one-letter code for one of the twenty amino acids, an arbitrary element (denoted by *x*), a set of different possible amino acids (e.g. [AGS]) or an arbitrary amino acid except specified aa (e.g. {DT}). Each symbol is then represented like a set: the set will contain only one element if the symbol at a given position is a specific amino acid; the set will contain more than one element if the symbol is represented by different letters in square brackets (a set of possible amino acids); the set will contain 20 elements (the 20 amino acids) if the symbol is represented by a *x *and, finally, the set will contain 20-*N *amino acids if the symbol is represented by curly brackets containing *N *letters. Then the *pair_score *is:(7)

where the numerator is the number of common amino acids between two sets (e.g. symbols) and the denominator is the product between the sizes of the two amino acids sets of the two corresponding symbols.

According to the Prosite language, the repetition of an element in the pattern is specified with a numerical value (or range) between parentheses, such that *x*(3) corresponds to *x *- *x *- *x *and *x*(1, 3) to *x *or *x *- *x *or *x *- *x *- *x*. When a range is specified inside the parentheses, there can be more 'instances' associated with the same pattern: in this case all instances from a given pattern are compared against all instances of another pattern. The similarity score between the two patterns is then the maximum score between all possible instances.

### Functional Annotation

• **UniProtKB features **PDB chains (or residues) and UniProtKB entries are mapped to each other through the PDBWS database [53] in order to obtain annotation directly at the residue level. The annotation is then mapped to each fragment.

• **Prosite annotation **Prosite annotation of clustering-derived sequence motifs is as described in the previous subsection.

• **Gene Ontology (GO) annotation **The enrichment of sequence motifs in some GO categories is done by first counting the number of hits of a motif against the Swiss-Prot-TM dataset and then retrieving the corresponding GO annotations from the GOA database at different levels of the hierarchy [54]. The hyper-geometric distribution is used to assess the significance of the enriched GO categories, in order to obtain the chance probability of observing a given functional category in the subset of sequences carrying a given motif. More specifically, p-values are calculated for a given GO term *t *and sequence motif *M *in the following way:(8)

where *G *is the total number of protein sequences in the Swiss-Prot-TM dataset; *C *is the number of transmembrane proteins annotated with the GO term *t*; *n *is the number of transmembrane sequences carrying the motif *M *and *k *is the number of sequences carrying the motif *M*, which are annotated with GO term *t*. This formula expresses the probability of observing at least *k *sequences from a functional category within a subset *n *carrying a given motif *M*. A critical value for the p-value is set to  (Bonferroni correction) with a threshold *α *= 0.01 and *N *= 24287 (number of different GO categories). Furthermore, the coverage *c *of a given GO term associated to a given motif:(9)

where *i *and *n *are defined as above.

• **Interaction interface annotation **The SCOPPI database [29], which classifies all the protein domain-domain interactions contained in the PDB, is used to check whether fragments in a structural class can be found at the interaction interface of protein complexes or at homomeric and heteromeric protein interfaces. The SCOPPI database contains information about residues belonging to a domain-domain interface, defined on the basis of geometric criteria, *i.e*. a domain residue is part of an interface if it is within 5 Å distance of another domain. This information is mapped on the members of a structural class defining a sequence motif. Furthermore, the whole PDB is screened to retrieve protein residues in contact with ligands or cofactors and this information is mapped on the derived sequence motifs.

• **Mutation analysis **For each motif and for each Swiss-Prot sequence where a certain motif matches, possible mutations in that sequence and in the range of amino acid positions from the motif start position to the motif stop position, are checked in literature by means of an automated text-mining approach. This approach is a rule- and regular expression-based protein point mutation retrieval pipeline for PubMed abstracts. It uses a named entity recognition algorithm for the identification of gene names-mutations co-occurrences in paper abstracts. Uniprot protein sequences for each identified gene are obtained and compared to the wild-type residues of the corresponding mutations. Whenever there is evidence that a given motif can be associated with a mutagenesis experiment described in literature, the PubMed reference describing the effect of the point mutation on the protein structure/function, and the mutation itself are included in the functional annotation of the motif.

### Propensities and P-values

To survey frequently occurring motifs in *α*-helical transmembrane proteins, we compute the occurrences of all motifs (4843 in total) in the Swiss-Prot-TM database. In order to identify overrepresented motifs in the Swiss-Prot-TM database, the expectation of occurrence and standard deviation for a pattern are calculated by randomly permuting the sequences in the database 100 times. An expectancy distribution is empirically generated by sampling the occurrences at random shuffling of the sequences. For expectation and p-value calculations, e.g. the probability of finding a certain number of occurrences of a motif after all sequences have been randomly permutated, the approach described in [55] is followed, by deriving a normal theoretical distribution of expectancy of each motif. The p-value for a given motif *M*, which occurs *N *times in the database, is the probability that *M *will occur *N *or more times. From the expectation value  relative to the occurrence of motif M we determine the odd ratio relative to the true occurrence value *N*_*M *_as: .

In order to assess the specificity of a given motif for transmembrane proteins, in contrast to globular proteins, the number of occurrences, *N*_*GLOB*_, of a given motif on a dataset of globular proteins derived from Swiss-Prot (Swiss-Prot-GLOB) is also calculated. A *false positive rate *number, defined as *N*_*GLOB *_/(*N*_*GLOB *_+ *N*_*M*_) is calculated. The significance of the enrichment of a given motif for transmembrane proteins is assessed by means of the p-value of the hyper-geometric distribution.

## Authors' contributions

AM wrote the paper. AM and AH conceived the study, wrote most of the programs, carried out data analysis and led the mathematical formulation of the algorithms. BV and KS wrote programs and participated in the mathematical formulation of some of the algorithms. AT provided help with literature review, biological interpretation of the results and review of the manuscript draft. CW helped with the motif data analysis and review of the manuscript draft. MS provided inputs in designing the study and reviewed the manuscript. All authors read and approved the final manuscript.

## Supplementary Material

Additional file 1 List of 58 non-redundant significant novel motifs and their basic structural/functional descriptions. Click here for file

Additional file 2 Statistics for both hydrogen bond- and backbone torsion angle-based clustering. Click here for file

Additional file 3 Examples of very well known structural motifs: 3_10_-helix and Schellmann motif. Click here for file

Additional file 4 Selection of the Prosite comparison cutoff. Click here for file

Additional file 5 Distributions of minimum and average resolution of clusters. Click here for file
