# Supplementary material for: Structural fragment clustering reveals novel structural and functional motifs in α-helical transmembrane proteins
Source: BMC Bioinformatics. 2010 Apr 26;11:204. doi: 10.1186/1471-2105-11-204 (PMC2876129; doi:10.1186/1471-2105-11-204)

Appendix A - Higly significant non redundant motifs

LEGEND

Structural characterisation: qualitative description of the main structural features of the motif and its location with respect to the lipid bilayer planes.
Function: enriched GO terms describing the putative function of the motif. When further information about the motif's function is available (e.g. from literature), it is also reported here.
Family: Pfam family/families where the motif hits. If the motif is specific to a certain family, the Pfam identifier is reported here; if the motif is found across more than four different families is labelled as *across families*.
Transmembrane protein abundance: it refers to the number of the motif's hits in all the transmembrane proteins from the Swiss-Prot database. If the number of hits is less than 100 the abundance is considered ***low***; if this number is between 100 and 500 the abundance is considered ***medium*** and if it is higher than 500 the abundance is considered ***high***.
Transmembrane protein specificity: it refers to the false positive rate associated to the motif, *i.e* number of the motif's hits in Swiss-Prot globular proteins divided by the total number of the motif's hit in the whole Swiss-Prot database. If the false positive rate is less than 10% the specificity is considered ***high***; if the false positive rate is between 10% and 40% the specificity is considered ***medium*** and if the false positive rate is greater than 40% the specificity is considered ***low***.
No of hits in Swiss-Prot: total number of hits of the motif in the Swiss-Prot database.
No of hits in Swiss-Prot-TM: number of hits of the motif in all transmembrane proteins in the Swiss-Prot database.
No of false positives: number of hits of the motif in all globular proteins in the Swiss-Prot database.
Interactions: information about the automated extraction of protein-protein interaction interfaces the motif is involved in from the SCOPPI database and ligand/cofactor/lipid/ion binding from the PDB database.
Mutations: information about the automated extraction of mutations from literature. When this information is available, the mutated residue position is reported, together with the Uniprot id of the sequence where the mutation was found, the organism's name and the Pubmed id of the article the mutation was extracted from.
Web logo: web logo of the motif.
Structural alignment: picture of the structural alignment of the fragments in the cluster corresponding to the motif.
Hydrogen bond pattern: schematic representation of the hydrogen bond pattern associated to the motif, when it is possible to derive such a conserved pattern. Each rectangular box in the picture corresponds to a given residue position in the the fragment. Highly conserved hydrogen bond patterns between main chain atoms (upper arcs in the picture) and side chain atoms (lower arcs in the picture) are shown by thicker arcs. Boxes corresponding to different residue positions are coloured on the basis of the conservetion score of a residue inside a cluster, ranging from red (highly conserved) to blue (weakly conserved). Dots of different colours in the boxes correspond to the Swiss-Prot feature annotation according to the legend in the bottom of the picture.
Backbone torsion angles: plot of the average values of Psi and Phi backbone angles for each residue position in the motif. Error bars are also shown. Each colour correspond to a different position in the fragments, according to the following scheme:
- residue position 1: red
- residue position 2: green
- residue position 3: blue
- residue position 4: brown
- residue position 5: dark green
- residue position 6: dark blue
- residue position 7: yellow
- residue position 8: magenta
- residue position 9: cyan
- residue position 10: grey
- residue position 11: military green
- residue position 12: violet
- residue position 13: pink
- residue position 14: dark yellow

Reentrant motif at the voltage gated chloride channel dimer interface A-[AS]-[FV]-[NR]-A-P-L-[AT]-G

Structural characterisation: Reentrant region
Function: voltage-gated chloride channel activity, chloride ion binding, antiporter activity, chloride transport
Family: PF00654 Voltage gated chloride channel
Transmembrane protein abundance: low, **Transmembrane protein specificity**: high
No of hits in Swiss-Prot: 23
No of hits in Swiss-Prot-TM: 23
No of false positives: 0 (0%)
Interactions: at the dimer interface
Mutations: no

WebLogo
Length 14

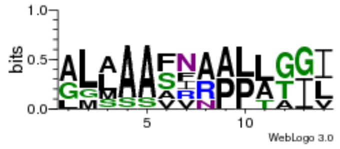

Structural Alignment

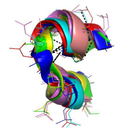

Hydrogen Bonds Pattern
Length 14

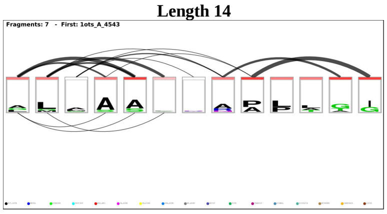

Backbone Torsion Angles
Length 14

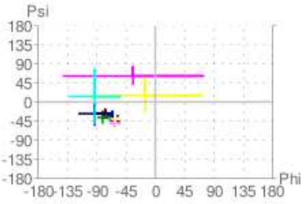

Membrane-water interface helix motif G-G-G-x-[NS]-x-[ILMV]-[AP]-[AIL]-[AG]

Structural characterisation: structured loop/interface helix on the extracellular side of the membrane
Function:
Family: across families
Transmembrane protein abundance: medium, **Transmembrane protein specificity**: medium
No of hits in Swiss-Prot: 47
No of hits in Swiss-Prot-TM: 31
No of false positives: 16 (34%)
Interactions: Flavin-Adenine dinucleotide (FAD) binding
Mutations: no

WebLogo
Length 13

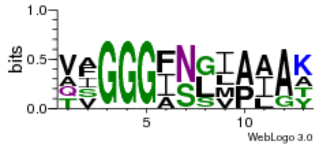

Structural Alignment

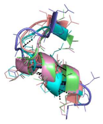

Hydrogen Bonds Pattern
Length 13

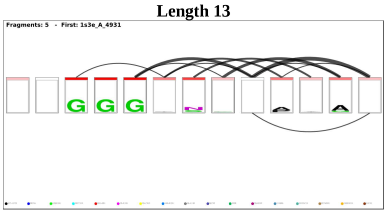

Backbone Torsion Angles
Length 13

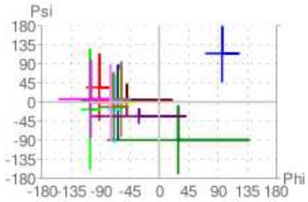

Across families membrane-water interface helix motif L-x-S-I-[GP]

Structural characterisation: interface helix, parallel to the membrane plane, on the extracellular side of the membrane
Function: convergent evolution, gating mechanism
Family: across families
Transmembrane protein abundance: high, **Transmembrane protein specificity**: medium
No of hits in Swiss-Prot: 5001
No of hits in Swiss-Prot-TM: 3002
No of false positives: 2001 (40%)
Interactions: cofactor binding
Mutations: no

WebLogo
Length 9

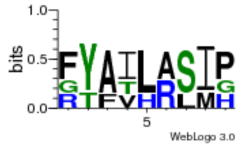

Structural Alignment

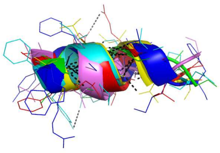

Hydrogen Bonds Pattern
Length 10

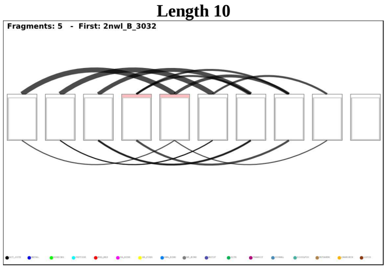

Backbone Torsion Angles
Length 10

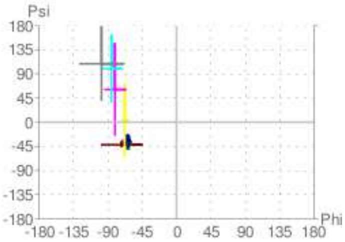

Across families motif at the end of helices G-F-x-A-[ATV]-x-[FW]

Structural characterisation: regular helix motif at the end of alpha-helices, close to the mebrane-water interface
Function:
Family: across families
Transmembrane protein abundance: high, **Transmembrane protein specificity**: medium
:**No of hits in Swiss-Prot**: 438
:**No of hits in Swiss-Prot-TM**: 265
:**No of false positives**: 173 (39.5%)
Interactions: ligand binding, protein-protein interaction interface
Mutations: no

WebLogo
Length 7

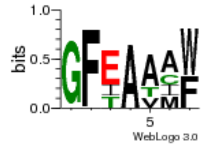

Structural Alignment

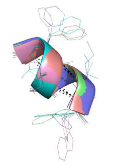

Hydrogen Bonds Pattern
Length 7

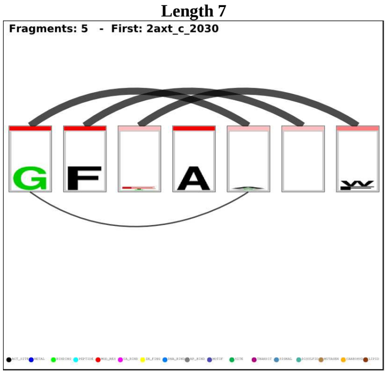

Backbone Torsion Angles
Length 7

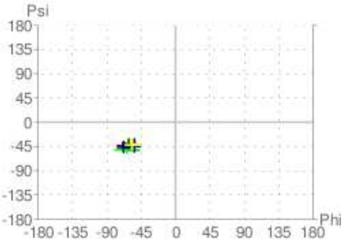

Across families motif at the end of helices F-[ASV]-L-x-[FWY]-L

Structural characterisation: regular helix motif at the end of alpha-helices, close to the mebrane-water interface
Function: Family: across families
Transmembrane protein abundance: high, **Transmembrane protein specificity**: medium
No of hits in Swiss-Prot: 1527
No of hits in Swiss-Prot-TM: 1099
No of false positives: 428 (28%)
Interactions: heme binding pocket, protein-protein interaction interface
Mutations: V93I - human - uniprot_id:P63252 - PMID:15922306, V93I - mouse - uniprot_id:P35561 - PMID:15922306

WebLogo
Length 8

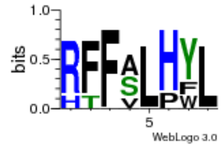

Structural Alignment

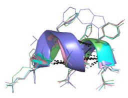

Hydrogen Bonds Pattern
Length 8

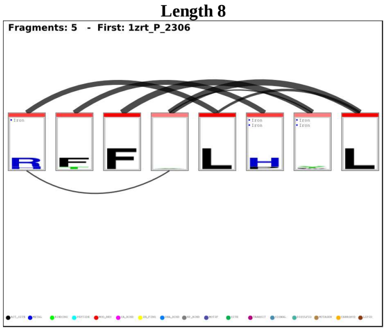

Backbone Torsion Angles
Length 8

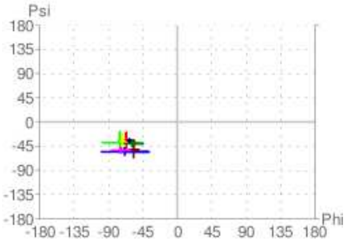

Across families motif at the end of helices L-F-L-x-[AV]-x-[FI]

Structural characterisation: regular helix motif at the end of alpha-helices, close to the mebrane-water interface
Function: cytochrome-c oxidase activity
Family: across families
Transmembrane protein abundance: high, **Transmembrane protein specificity**: medium
No of hits in Swiss-Prot: 649
No of hits in Swiss-Prot-TM: 463
No of false positives: 186 (28.6%)
Interactions: ligand binding
Mutations: no

WebLogo
Length 8

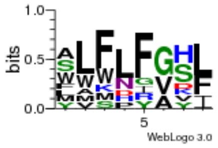

Structural Alignment

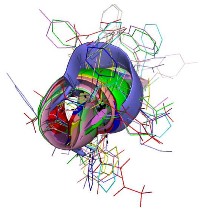

Hydrogen Bonds Pattern
Length 8

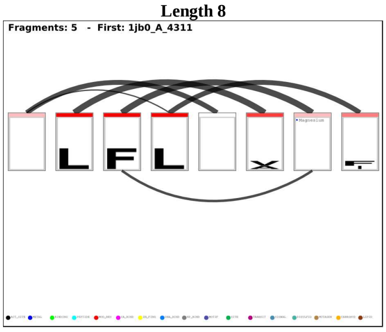

Backbone Torsion Angles
Length 8

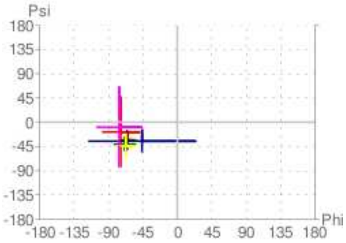

Putative helix-helix packing motif at the end of alpha-helices A-F-[MV]-G-[FY]

Structural characterisation: regular helix motif at the end of alpha-helices, close to the mebrane-water interface
Function: helix-helix packing
Family: across families
Transmembrane protein abundance: high, **Transmembrane protein specificity**: high
No of hits in Swiss-Prot: 1782
No of hits in Swiss-Prot-TM: 1691
No of false positives: 91 (5.1%)
Interactions: lipid interactions
Mutations: no

WebLogo
Length 8

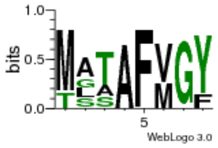

Structural Alignment

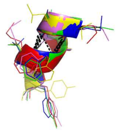

Hydrogen Bonds Pattern
Length 8

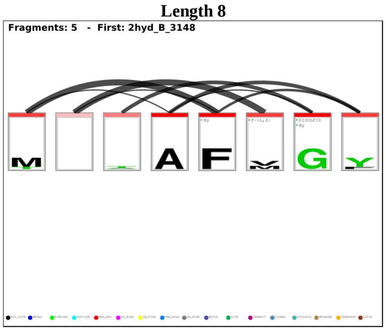

Backbone Torsion Angles
Length 8

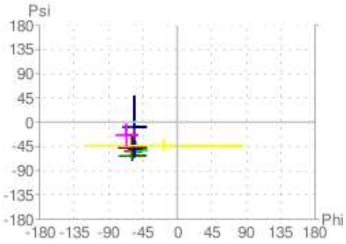

Across families motif at the end of helices A-[FV]-[LMV]-G-Y-[CV]

Structural characterisation: regular helix motif at the end of alpha-helices, close to the mebrane-water interface
Function: cytochrome-c oxidase activity, electron transport
Family: across families, mostly PF00510 cytochrome c oxidase
Transmembrane protein abundance: medium, **Transmembrane protein specificity**: high
No of hits in Swiss-Prot: 108
No of hits in Swiss-Prot-TM: 103
No of false positives: 5 (4.6%)
Interactions: protein-protein interaction interface
Mutations: no

WebLogo
Length 8

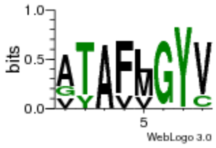

Structural Alignment

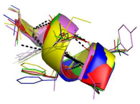

Hydrogen Bonds Pattern
Length 8

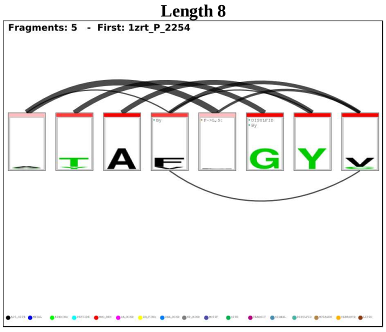

Backbone Torsion Angles
Length 8

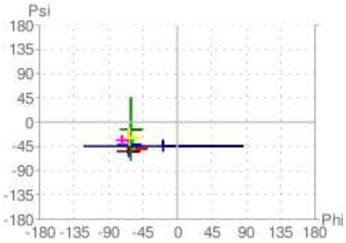

Heme binding pocket motif in cytochrome b F-[FY]-[AS]-[IL]-x(2)-L-[LV]-P

Structural characterisation: regular helix motif at the end of alpha-helices
Function: oxidoreductase activity, electron transport
Family: PF00032 Cytochrome b (C-terminal)
Transmembrane protein abundance: medium, **Transmembrane protein specificity**: high
No of hits in Swiss-Prot: 178
No of hits in Swiss-Prot-TM: 176
No of false positives: 2 (1.1%)
Interactions: heme binding, protein-protein interaction interface
Mutations: no

WebLogo
Length 9

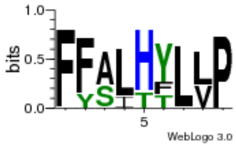

Structural Alignment

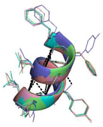

Hydrogen Bonds Pattern
Length 9

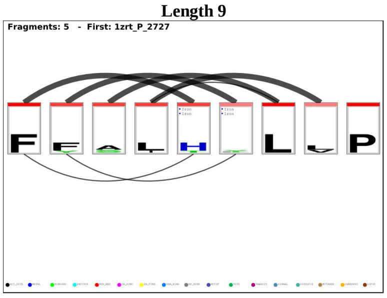

Backbone Torsion Angles
Length 9

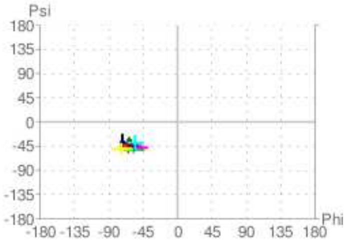

Helical motif in cytochrome b R-F-F-[AS]-[FL]-H-x(1,2)-L

Structural characterisation: regular helix motif
Function: electron transport, oxidoreductase activity, iron ion binding
Family: PF00033 Cytochrome b (N-terminal)
Transmembrane protein abundance: high, **Transmembrane protein specificity**: high
No of hits in Swiss-Prot: 1527
No of hits in Swiss-Prot-TM: 1526
No of false positives: 1 (0.06%)
Interactions: heme binding, iron ion binding, protein-protein interaction interface
Mutations: no

WebLogo
Length 9

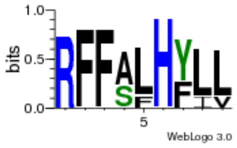

Structural Alignment

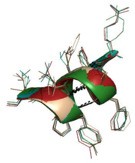

Hydrogen Bonds Pattern
Length 9

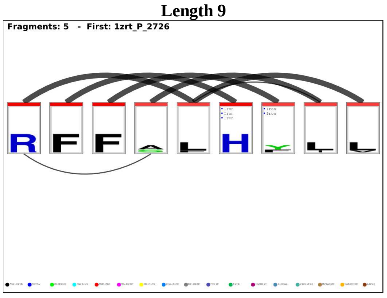

Backbone Torsion Angles
Length 9

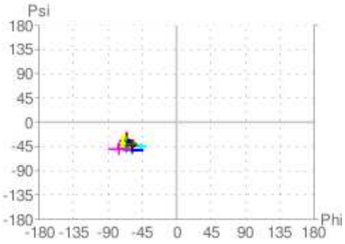

Helical motif in cytochrome b T-[IL]-[NQT]-R-F-F-[AS]-[FL]-H

Structural characterisation: regular helix motif
Function: electron transport, oxidoreductase activity, iron ion binding
Family: PF00033 Cytochrome b (N-terminal)
Transmembrane protein abundance: high, **Transmembrane protein specificity**: high
No of hits in Swiss-Prot: 1562
No of hits in Swiss-Prot-TM: 1561
No of false positives: 1 (0.06%)
Interactions: heme binding, iron ion binding, protein-protein interaction interface
Mutations: no

WebLogo
Length 9

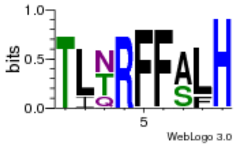

Structural Alignment

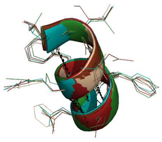

Hydrogen Bonds Pattern
Length 9

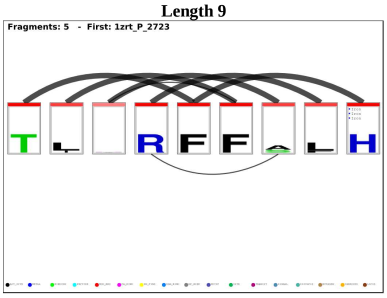

Backbone Torsion Angles
Length 9

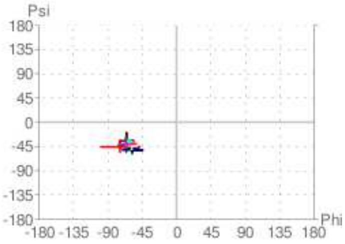

Helical motif in cytochrome b R-F-F-[AS]-[FL]-H-[FY]

Structural characterisation: regular helix motif
Function: electron transport, oxidoreductase activity, iron ion binding
Family: PF00033 Cytochrome b (N-terminal)
Transmembrane protein abundance: high, **Transmembrane protein specificity**: high
No of hits in Swiss-Prot: 1566
No of hits in Swiss-Prot-TM: 1565
No of false positives: 1 (0.06%)
Interactions: heme binding, iron ion binding, protein-protein interaction interface
Mutations: no

WebLogo
Length 9

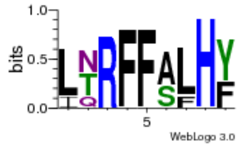

Structural Alignment

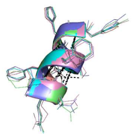

Hydrogen Bonds Pattern
Length 9

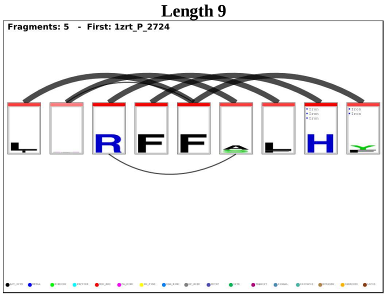

Backbone Torsion Angles
Length 9

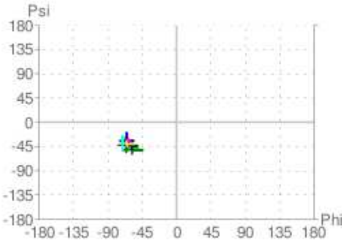

Across families motif at the end of helices G-F-x-A-[ATV]-x-[FW]

Structural characterisation: regular helix motif
Function: electron transport, photosynthesis, transferring electrons within the cyclic electron transport pathway of photosynthesis activity
Family: across families, mostly PF00032 Cytochrome b (C-terminal)
Transmembrane protein abundance: medium, **Transmembrane protein specificity**: medium
No of hits in Swiss-Prot: 130
No of hits in Swiss-Prot-TM: 107
No of false positives: 23 (17.7%)
Interactions: ligand binding
Mutations: no

WebLogo
Length 7

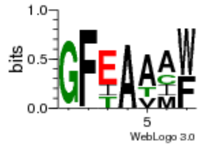

Structural Alignment

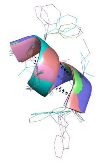

Hydrogen Bonds Pattern
Length 7

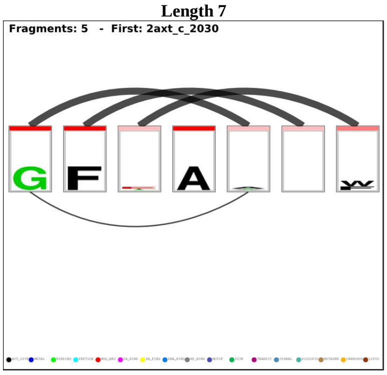

Backbone Torsion Angles
Length 7

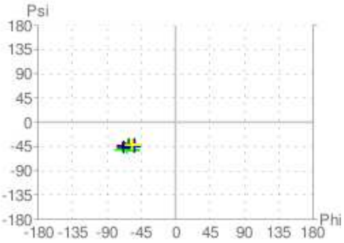

Across families Trp/Phe kink motif W-L-F-[ST]

Structural characterisation: irregular alpha-helix/kink in the middle of transmembrane alpha-helices due to the aromatic residues Trp and Phe
Function:
Family: across families
Transmembrane protein abundance: high, **Transmembrane protein specificity**: medium
No of hits in Swiss-Prot: 772
No of hits in Swiss-Prot-TM: 479
No of false positives: 293 (38%)
Interactions: no
Mutations: no

WebLogo
Length 5

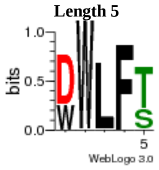

Structural Alignment

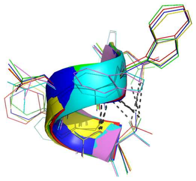

Hydrogen Bonds Pattern
Length 5

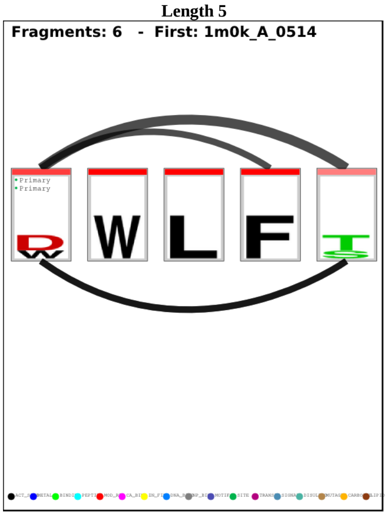

Backbone Torsion Angles
Length 5

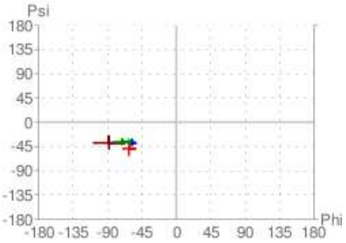

Retinal binding pocket motif in bacteriorhodopsin Y-P-[FIV]-[ILV]-W-[IL]-[ILV]-G-[PST]

Structural characterisation: regular helix motif
Function: photoreceptor activity, ion channel activity, proton transport, phototransduction
Family: PF01036 Bacteriorhodopsin
Transmembrane protein abundance: low, **Transmembrane protein specificity**: high
No of hits in Swiss-Prot: 9
No of hits in Swiss-Prot-TM: 9
No of false positives: 0 (0%)
Interactions: retinal binding pocket
Mutations: no

WebLogo
Length 9

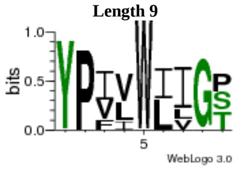

Structural Alignment

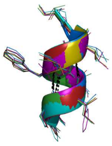

Hydrogen Bonds Pattern
Length 9

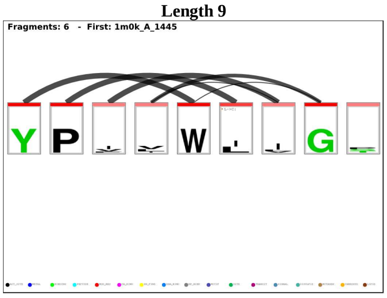

Backbone Torsion Angles
Length 9

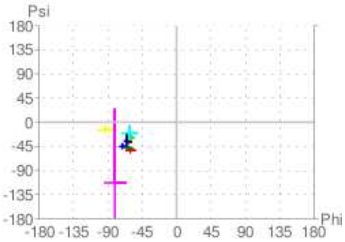

Retinal binding pocket motif in bacteriorhodopsin Y-P-[IV]-[LV]-W-x(0,1)-I-x(0,1)-G-[PST]-[ES]

Structural characterisation: regular helix motif
Function: photoreceptor activity, ion channel activity, proton transport, phototransduction
Family: PF01036 Bacteriorhodopsin
Transmembrane protein abundance: low, **Transmembrane protein specificity**: high
No of hits in Swiss-Prot: 7
No of hits in Swiss-Prot-TM: 7
No of false positives: 0 (0%)
Interactions: retinal binding pocket
Mutations: no

WebLogo
Length 10

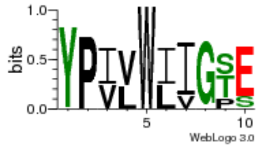

Structural Alignment

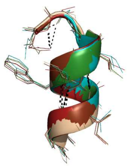

Hydrogen Bonds Pattern
Length 10

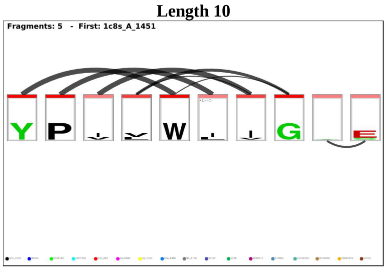

Backbone Torsion Angles
Length 10

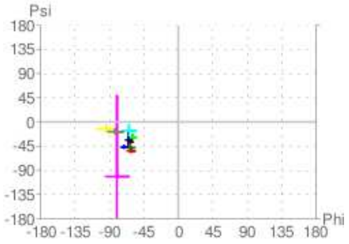

Heme binding pocket motif in cytochrome b R-x-[ILM]-H-A-N-G-A

Structural characterisation: regular helix motif at the end of alpha-helices
Function: iron ion binding, oxidoreductase activity, electron transport
Family: PF00033 Cytochrome b
Transmembrane protein abundance: high, **Transmembrane protein specificity**: high
No of hits in Swiss-Prot: 1527
No of hits in Swiss-Prot-TM: 1525
No of false positives: 2 (0.13%)
Interactions: heme binding, iron ion binding
Mutations: no

WebLogo
Length 10

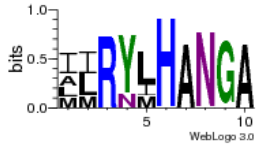

Structural Alignment

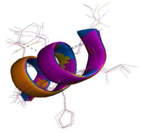

Hydrogen Bonds Pattern
Length 10

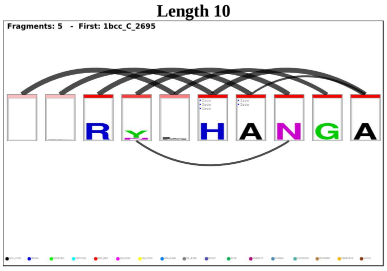

Backbone Torsion Angles
Length 10

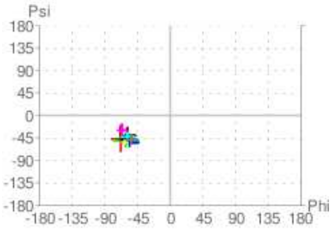

Across families tilted alpha-helix motif G-[FILM]-[AIL]-L-F-I-[FIM]

Structural characterisation: tilted alpha-helix at the membrane water interface
Function: structural motif, probably important for interactions with lipids
Family: across families
Transmembrane protein abundance: medium, **Transmembrane protein specificity**: high
No of hits in Swiss-Prot: 83
No of hits in Swiss-Prot-TM: 79
No of false positives: 4 (4.8%)
Interactions: lipid interaction
Mutations: no

WebLogo
Length 9

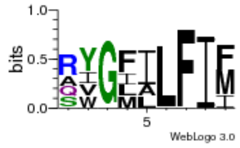

Structural Alignment

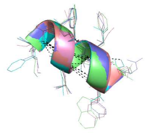

Hydrogen Bonds Pattern
Length 9

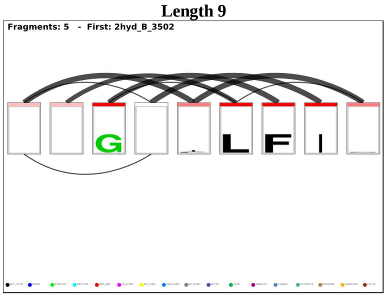

Backbone Torsion Angles
Length 9

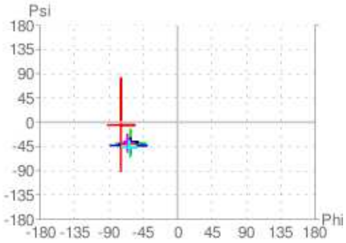

Retinal binding pocket motif in bacteriorhodopsin P-[IV]-[LV]-W-x(0,1)-I-x(0,1)-G-[PST]-[ES]

Structural characterisation: regular helix motif
Function: photoreceptor activity, ion channel activity, proton transport, phototransduction
Family: PF01036 Bacteriorhodopsin
Transmembrane protein abundance: low, **Transmembrane protein specificity**: high
No of hits in Swiss-Prot: 7
No of hits in Swiss-Prot-TM: 7
No of false positives: 0 (0%)
Interactions: retinal binding pocket
Mutations: no

WebLogo
Length 9

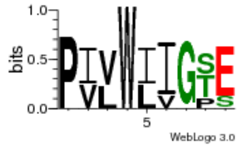

Structural Alignment

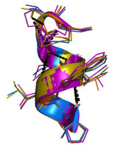

Hydrogen Bonds Pattern
Length 9

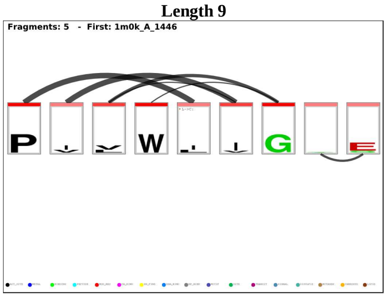

Backbone Torsion Angles
Length 9

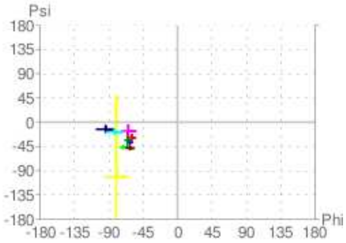

Irregular helix motif in cytochrome b G-[IL]-[FLV]-[LM]-A-M-H-Y-[ST]

Structural characterisation: irregular helix at the membrane-water interface
Function:
Family: PF00033 Cytochrome b (N-terminal)
Transmembrane protein abundance: high, **Transmembrane protein specificity**: high
No of hits in Swiss-Prot: 1472
No of hits in Swiss-Prot-TM: 1471
No of false positives: 1 (0.07%)
Interactions: heme binding, protein-protein interaction interface
Mutations: no

WebLogo
Length 9

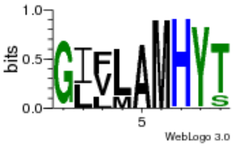

Structural Alignment

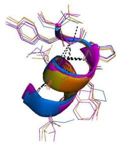

Hydrogen Bonds Pattern
Length 9

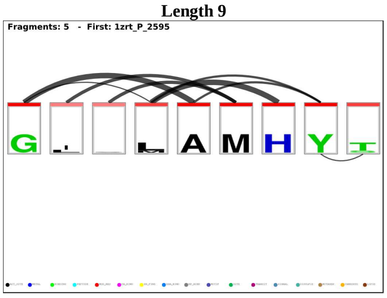

Backbone Torsion Angles
Length 9

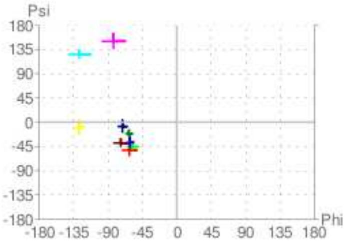

Across families loop motif L-G-[ILM]-G-x-H-x-[AP]

Structural characterisation: irregular helix/loop at the membrane-water interface
Function: photosynthesis, light reaction, metal ion binding, light-harvesting complex
Family: across families, mostly PF00124
Transmembrane protein abundance: low, **Transmembrane protein specificity**: medium
No of hits in Swiss-Prot: 33
No of hits in Swiss-Prot-TM: 23
No of false positives: 10 (30%)
Interactions: ligand/lipid binding
Mutations: no

WebLogo
Length 9

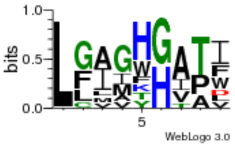

Structural Alignment

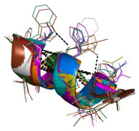

Hydrogen Bonds Pattern
Length 9

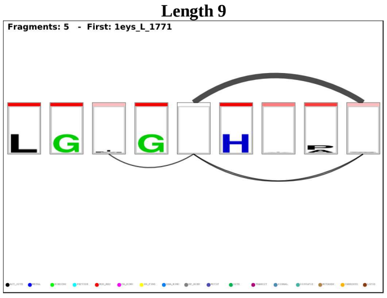

Backbone Torsion Angles
Length 9

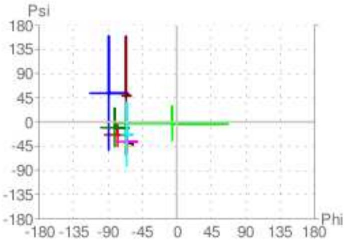

Across families loop motif G-[AIP]-x(2)-[ANV]-x-F-F-G-[IV]

Structural characterisation: irregular loop, end of helix motif at the membrane-water interface
Function: structural motif, interaction with lipids polar heads
Family: across the superfamilies CL0086 FAD-linked oxidoreductases, CL0062 APC superfamily
Transmembrane protein abundance: low, **Transmembrane protein specificity**: high
No of hits in Swiss-Prot: 25
No of hits in Swiss-Prot-TM: 24
No of false positives: 1 (4%)
Interactions: ligand binding
Mutations: no

WebLogo
Length 10

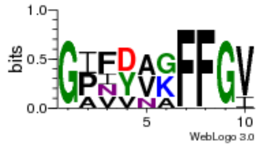

Structural Alignment

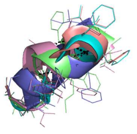

Hydrogen Bonds Pattern
Length 10

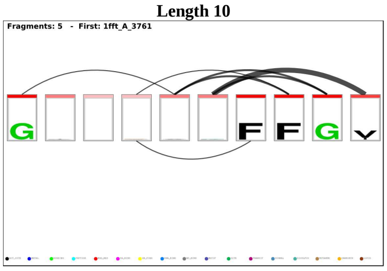

Backbone Torsion Angles
Length 10

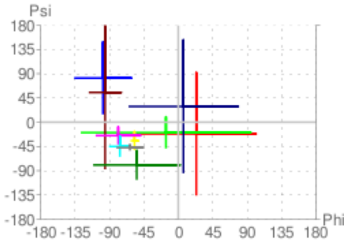

Across families helix motif G-[AIP]-x(2)-[ANV]-x-F-F-G-[IV]

Structural characterisation: regular helix at the membrane-water interface
Function: structural motif, interaction with lipids polar heads
Family: across families
Transmembrane protein abundance: medium, **Transmembrane protein specificity**: medium
No of hits in Swiss-Prot: 97
No of hits in Swiss-Prot-TM: 70
No of false positives: 27 (27.8%)
Interactions: ligand binding
Mutations: no

WebLogo
Length 10

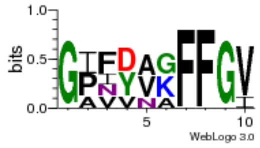

Structural Alignment

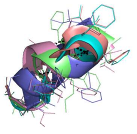

Hydrogen Bonds Pattern
Length 10

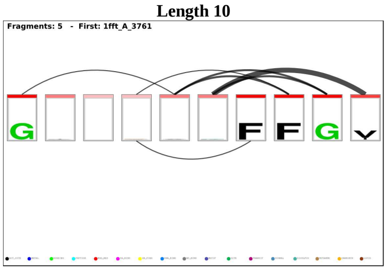

Backbone Torsion Angles
Length 10

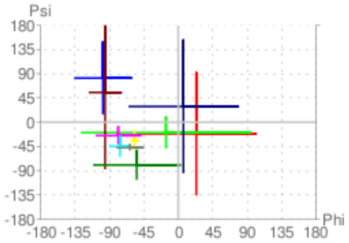

Tilted alpha-helix motif in cytochrome b
P-N-K-L-[GL]-x(2)-[IL]-[AL]-[ILM]

Structural characterisation: tilted alpha-helix at the membrane water interface
Function: mitochondrial electron transport chain, oxidoreductase activity, electron transport
Family: PF00032 Cytochrome b (C-terminal)
Transmembrane protein abundance: high, **Transmembrane protein specificity**: high
No of hits in Swiss-Prot: 1579
No of hits in Swiss-Prot-TM: 1579
No of false positives: 0 (0%)
Interactions: ligand binding, protein-protein interaction interface
Mutations: no

WebLogo
Length 10

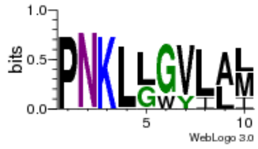

Structural Alignment

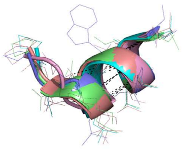

Hydrogen Bonds Pattern
Length 10

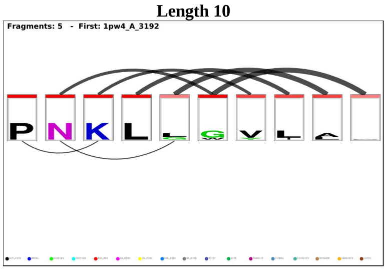

Backbone Torsion Angles
Length 10

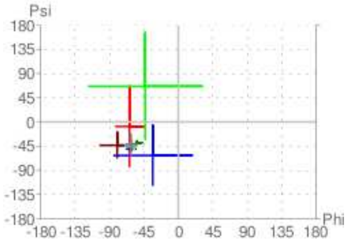

Across families membrane-water interface helix motif
R-[EQ]-x-E-x(2,3)-R-x(0,1)-L-G

Structural characterisation: interface helix, parallel to the membrane plane, on the cytoplasmic side of the membrane
Function: convergent evolution, putative gating mechanism
Family: across families
Transmembrane protein abundance: medium, **Transmembrane protein specificity**: medium
No of hits in Swiss-Prot: 194
No of hits in Swiss-Prot-TM: 121
No of false positives: 73 (37.6%)
Interactions: cofactor binding
Mutations: no

WebLogo
Length 10

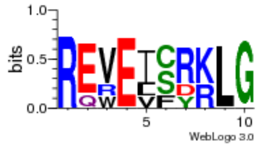

Structural Alignment

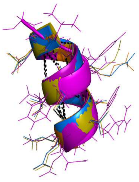

Hydrogen Bonds Pattern
Length 10

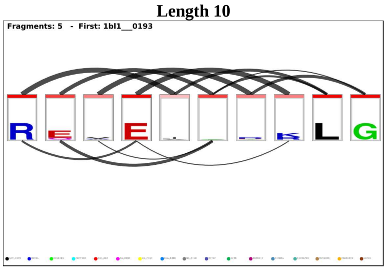

Backbone Torsion Angles
Length 10

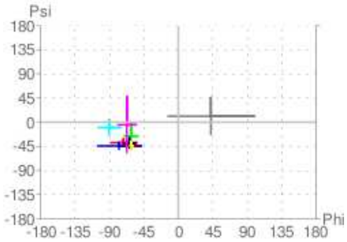

Helical motif in cytochrome b T-[IL]-[NQT]-R-F-F-[AS]-[FL]-H-[FY]

Structural characterisation: regular helix motif
Function: electron transport, oxidoreductase activity, iron ion binding
Family: PF00033 Cytochrome b (N-terminal)
Transmembrane protein abundance: high, **Transmembrane protein specificity**: high
No of hits in Swiss-Prot: 1556
No of hits in Swiss-Prot-TM: 1555
No of false positives: 1 (0.06%)
Interactions: heme binding, iron ion binding, protein-protein interaction interface
Mutations: no

WebLogo
Length 10

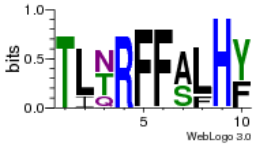

Structural Alignment

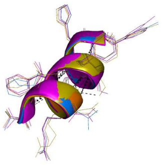

Hydrogen Bonds Pattern
Length 10

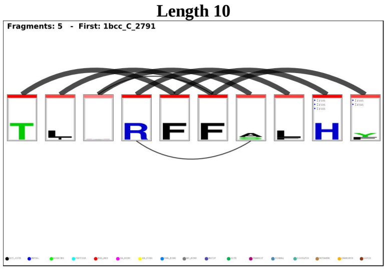

Backbone Torsion Angles
Length 10

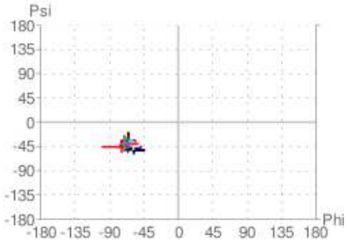

Helical motif in cytochrome b R-F-F-[AS]-[FL]-H-x(1,2)-L-x(0,1)-P

Structural characterisation: regular helix motif
Function: electron transport, oxidoreductase activity, iron ion binding
Family: PF00033 Cytochrome b (N-terminal)
Transmembrane protein abundance: high, **Transmembrane protein specificity**: high
No of hits in Swiss-Prot: 1525
No of hits in Swiss-Prot-TM: 1524
No of false positives: 1 (0.06%)
Interactions: heme binding, iron ion binding, protein-protein interaction interface
Mutations: no

WebLogo
Length 10

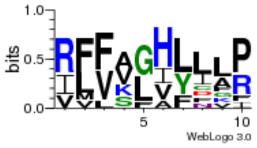

Structural Alignment

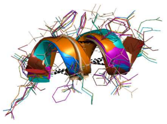

Hydrogen Bonds Pattern
Length 10

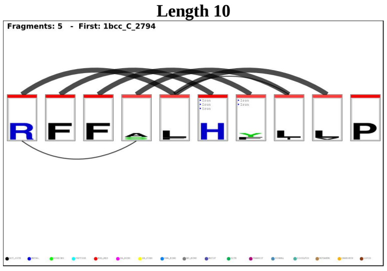

Backbone Torsion Angles
Length 10

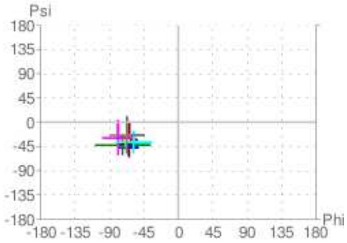

Structured loop motif in cytochrome b A-F-[LMV]-G-Y-x(0,2)-V-x(1,3)-G-Q-M-S

Structural characterisation: structured loop/irregular 3/10 helix on the cytoplasmic side of the membrane
Function: iron ion binding, oxidoreductase activity, mitochondrial electron transport chain
Family: PF00033 Cytochrome b (N-terminal)
Transmembrane protein abundance: high, **Transmembrane protein specificity**: high
No of hits in Swiss-Prot: 1560
No of hits in Swiss-Prot-TM: 1558
No of false positives: 2 (0.13%)
Interactions: heme binding, stigmatellin binding
Mutations: F129L - yeast - uniprot_id:P00163 - PMID:15912560, C133Y - yeast - uniprot_id:P00163 - PMID:8393450

WebLogo
Length 13

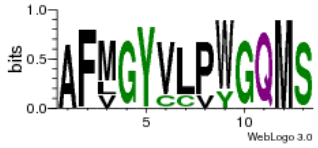

Structural Alignment

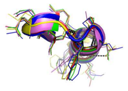

Hydrogen Bonds Pattern
Length 13

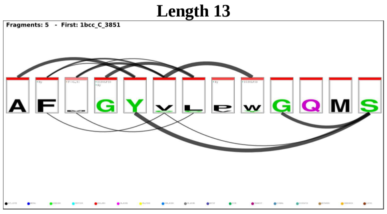

Backbone Torsion Angles
Length 13

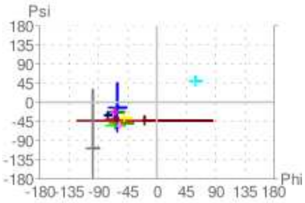

Across families tilted helix motif F-F-G-V-x-[AGT]-[FIM]

Structural characterisation: tilted alpha-helix at the membrane-water interface
Function: bacteriochlorophyll binding, light-harvesting complex, electron transport
Family: across families, mostly PF00033 Cytochrome b, PF00124 Photosynthetic reaction center
Transmembrane protein abundance: low, **Transmembrane protein specificity**: medium
No of hits in Swiss-Prot: 27
No of hits in Swiss-Prot-TM: 23
No of false positives: 4 (14.8%)
Interactions: lipid binding
Mutations: no

WebLogo
Length 12

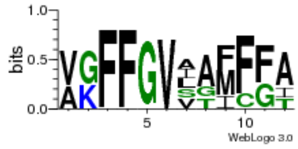

Structural Alignment

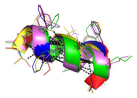

Hydrogen Bonds Pattern
Length 12

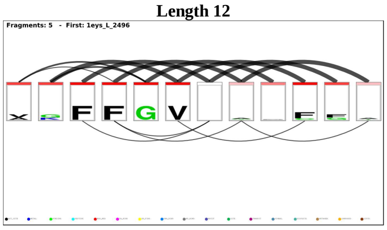

Backbone Torsion Angles
Length 12

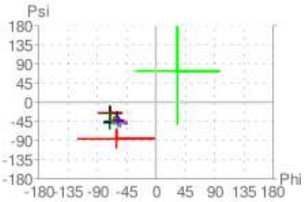

Loop motif in cytochrome b F-F-G-V-x-[AGT]-[FIM]

Structural characterisation: structured loop connecting two alpha-helices
Function: metal ion binding, oxidoreductase activity, mitochondrial electron transport chain
Family: PF00033 Cytochrome b (N-terminal)
Transmembrane protein abundance: high, **Transmembrane protein specificity**: high
No of hits in Swiss-Prot: 911
No of hits in Swiss-Prot-TM: 909
No of false positives: 2 (0.21%)
Interactions: heme binding
Mutations: no

WebLogo
Length 12

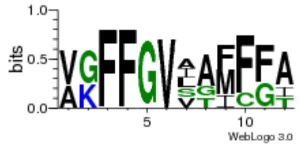

Structural Alignment

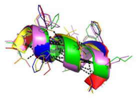

Hydrogen Bonds Pattern
Length 12

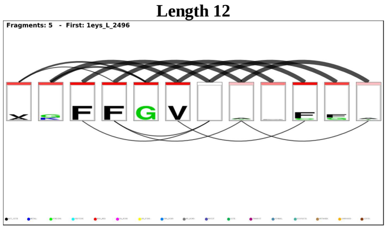

Backbone Torsion Angles
Length 12
Sample Chart

Proline kink motif in cytochrome c and quinol oxidases G-H-P-x-V-Y-[FI]

Structural characterisation: kink in the middle of transmembrane helices
Function: oxidoreductase, hydrogen ion transport
Family: PF00115 Cytochrome C and Quinol oxidase polypeptide I
Transmembrane protein abundance: high, **Transmembrane protein specificity**: high
No of hits in Swiss-Prot: 219
No of hits in Swiss-Prot-TM: 210
No of false positives: 9 (4.1%)
Interactions: copper binding
Mutations: no

WebLogo
Length 8

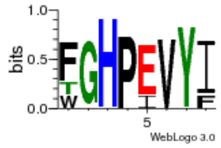

Structural Alignment

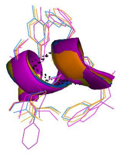

Hydrogen Bonds Pattern
Length 8

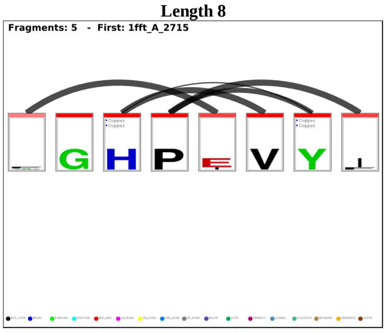

Backbone Torsion Angles
Length 8

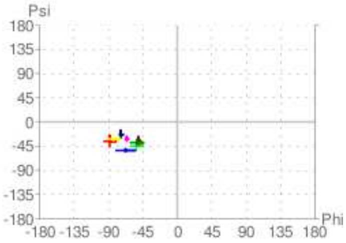

Proline kink motif in bacteriorhodopsins V-L-W-x-[AG]-Y-P

Structural characterisation: kink in the middle of transmembrane helices
Function: photoreceptor activity, ion transport, protein-chromophore linkage
Family: PF01036 Bacteriorhodopsin
Transmembrane protein abundance: low, **Transmembrane protein specificity**: high
No of hits in Swiss-Prot: 14
No of hits in Swiss-Prot-TM: 14
No of false positives: 0 (0%)
Interactions: retinal binding pocket
Mutations: no

WebLogo
Length 8

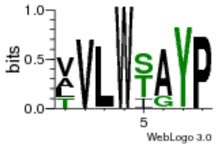

Structural Alignment

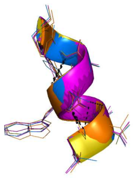

Hydrogen Bonds Pattern
Length 8

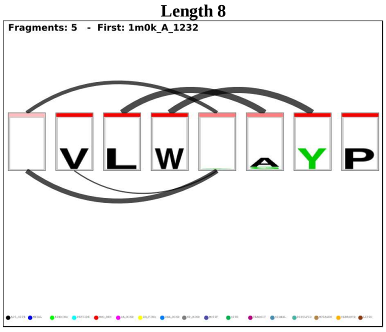

Backbone Torsion Angles
Length 8

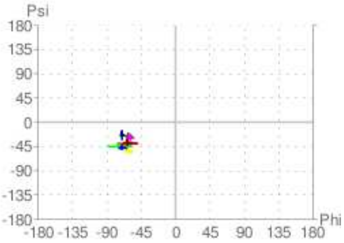

Across families Reentrant motif L-[AT]-G-[FI]-[AILV]-x-[IPV]-[IL]

Structural characterisation: Reentrant region
Function:
Family: across families, mostly PF00654 voltage gated chloride channels, PF00909 ammonium transporters, PF01554 multidrug resistant protein mdtk
Transmembrane protein abundance: medium, **Transmembrane protein specificity**: medium
No of hits in Swiss-Prot: 115
No of hits in Swiss-Prot-TM: 82
No of false positives: 33 (28.7%)
Interactions: protein-protein interaction interface
Mutations: no

WebLogo
Length 8

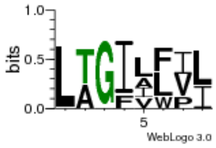

Structural Alignment

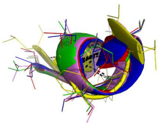

Hydrogen Bonds Pattern
Length 8

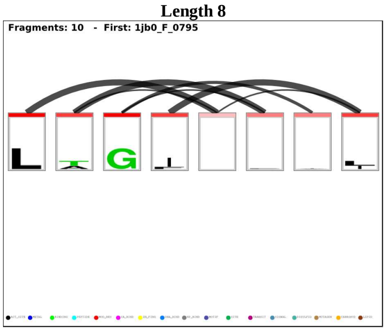

Backbone Torsion Angles
Length 8

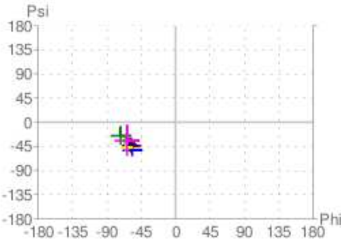

Retinal binding motif in bacterial opsins L-[DP]-[FLV]-x-[AST]-K-V-G-F-[GS]

Structural characterisation: irregular alpha-helix, pi-bulge characterised by main chain hydrogen bonds in positions i, i+5
Function: photoreceptor activity, ion channel activity, proton transport, phototransduction
Family: PF01036 Bacteriorhodopsin
Transmembrane protein abundance: low, **Transmembrane protein specificity**: high
No of hits in Swiss-Prot: 8
No of hits in Swiss-Prot-TM: 8
No of false positives: 0 (0%)
Interactions: retinal binding pocket
Mutations: no

WebLogo
Length 10

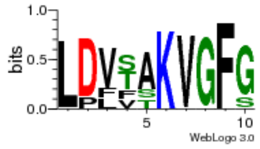

Structural Alignment

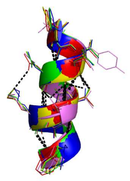

Hydrogen Bonds Pattern
Length 10
hydrogenbonds

Backbone Torsion Angles
Length 10

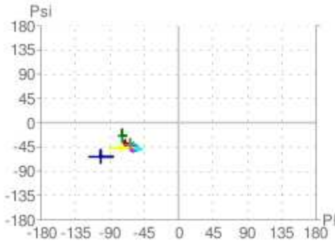

Heme packing motif in cytochrome b L-[IMV]-x-Q-I-[LV]-T-G-[IL]

Structural characterisation: membrane embedded regular alpha-helix
Function: metal ion binding, oxidoreductase activity, mitochondrial electron transport chain
Family: PF00033 Cytochrome b (N-terminal)
Transmembrane protein abundance: high, **Transmembrane protein specificity**: high
No of hits in Swiss-Prot: 873
No of hits in Swiss-Prot-TM: 872
No of false positives: 1 (0.11%)
Interactions: heme binding pocket
Mutations: no

WebLogo
Length 10

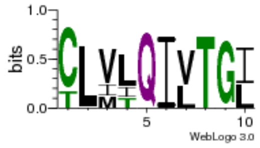

Structural Alignment

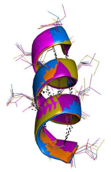

Hydrogen Bonds Pattern
Length 10

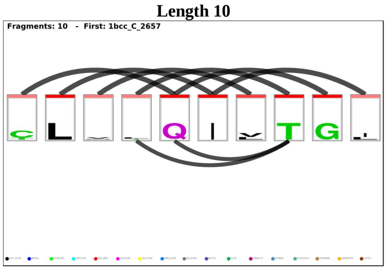

Backbone Torsion Angles
Length 10

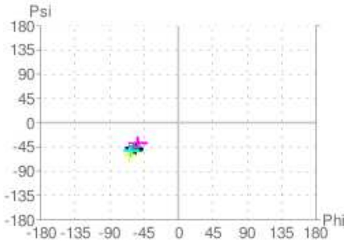

Loop motif in cytochrome c and quinol oxidases P-[AS]-G-G-G-D-P-[IV]

Structural characterisation: structured loop in the periplasmic spaceof Gram-negative bacteria
Function: copper ion binding, cytochrome-c oxidase activity, electron transport, aerobic respiration
Family: PF00115 Cytochrome c and Quinol oxidases
Transmembrane protein abundance: medium, **Transmembrane protein specificity**: high
No of hits in Swiss-Prot: 127
No of hits in Swiss-Prot-TM: 127
No of false positives: 0 (0%)
Interactions: cytochrome c
Mutations: no

WebLogo
Length 12

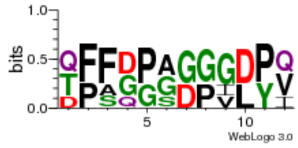

Structural Alignment

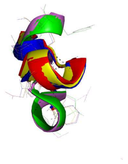

Hydrogen Bonds Pattern
Length 12

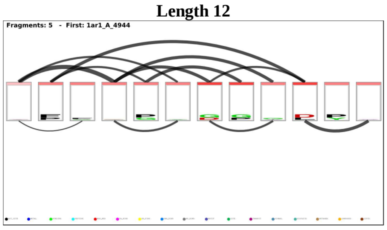

Backbone Torsion Angles
Length 12

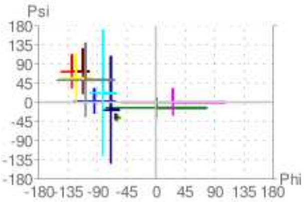

Loop motif in cytochrome b P-[DN]-x-L-G-[DH]-P-[DE]

Structural characterisation: structured loop on the cytoplasmic side of the membrane
Function: electron transport, oxidoreductase activity, iron ion binding
Family: PF000333 Cytochrome b (N-terminal)
Transmembrane protein abundance: high, **Transmembrane protein specificity**: high
No of hits in Swiss-Prot: 1481
No of hits in Swiss-Prot-TM: 1481
No of false positives: 0 (0%)
Interactions: interaction interface between subunit III and cytochrome c1
Mutations: no

WebLogo
Length 13

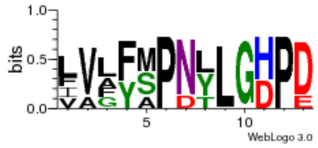

Structural Alignment

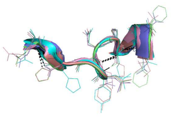

Hydrogen Bonds Pattern
Length 13

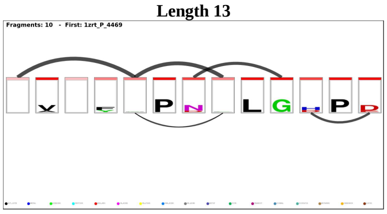

Backbone Torsion Angles
Length 13

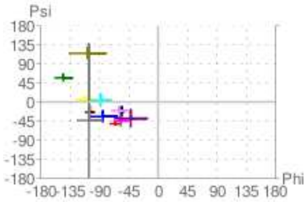

Stigmatellin binding loop motif in cytochrome b
T-A-F-[LMV]-G-Y-x(0,2)-V-x(1,3)-G

Structural characterisation: irregular helix/loop at the mebrane-water interface
Function: oxidoreductase activity, iron ion binding, mitochondrial electron transport chain
Family: PF00033 Cytochrome b (N-terminal)
Transmembrane protein abundance: high, **Transmembrane protein specificity**: high
No of hits in Swiss-Prot: 1555
No of hits in Swiss-Prot-TM: 1553
No of false positives: 2 (0.13%)
Interactions: heme binding, stigmatellin binding
Mutations: F129L - yeast - uniprot_id:P00163 - PMID:15912560, C133Y - yeast - uniprot_id:P00163 - PMID:8393450

WebLogo
Length 13

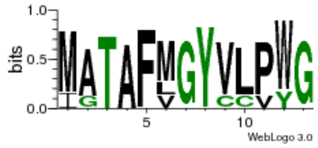

Structural Alignment

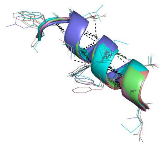

Hydrogen Bonds Pattern
Length 13

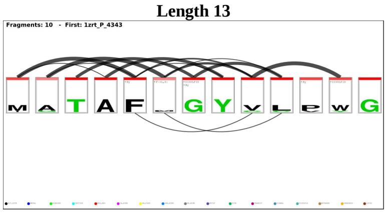

Backbone Torsion Angles
Length 13

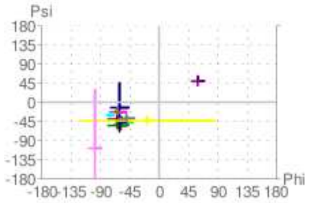

Across families helix-helix packing motif
V-G-x-L-[ACTV]-[AGT]-[IL]-[AGV]

Structural characterisation: membrane-embedded right-handed regular helix
Function: ion channel activity, metal ion binding, transport
Family: across families, mostly CL0062 APC superfamily and CL0030 Ion channels
Transmembrane protein abundance: high, **Transmembrane protein specificity**: medium
No of hits in Swiss-Prot: 285
No of hits in Swiss-Prot-TM: 214
No of false positives: 71 (24.9%)
Interactions: intra-protein helix-helix interaction interface
Mutations: no

WebLogo
Length 10

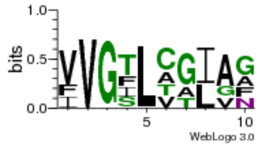

Structural Alignment

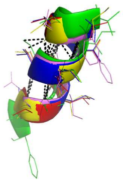

Hydrogen Bonds Pattern
Length 10

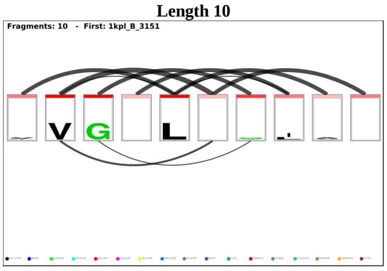

Backbone Torsion Angles
Length 10

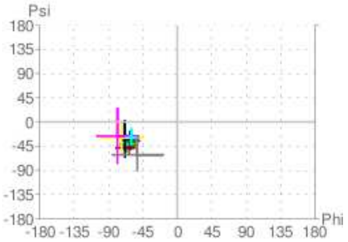

heme/copper binding site in cytochrome c and quinol oxidases W-x(2)-G-H-P-x-V-Y-[FI]-x-[ILV]-L

Structural characterisation: helix with a proline kink in the middle
Function: cytochrome-c oxidase activity, copper ion binding, electron transport, iron ion binding
Family: PF00115 Cytochrome C and Quinol oxidases
Transmembrane protein abundance: medium, **Transmembrane protein specificity**: high
No of hits in Swiss-Prot: 174
No of hits in Swiss-Prot-TM: 174
No of false positives: 0 (0%)
Interactions: heme binding, copper binding (His residue)
Mutations: no

WebLogo
Length 13

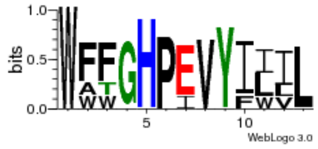

Structural Alignment

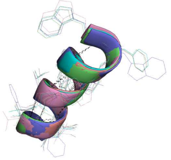

Hydrogen Bonds Pattern
Length 13

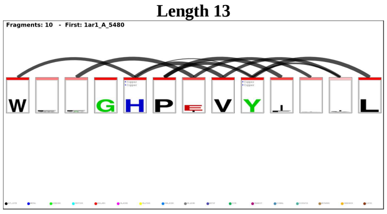

Backbone Torsion Angles
Length 13

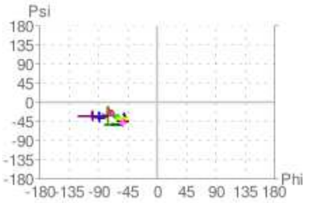

Tilted alpha-helix motif in photosystem II L-x-[AIV]-x-[HW]-G-A-T

Structural characterisation: tilted alpha-helix at the membrane-water interface
Function: chloroplast, photosynthesis, metal ion binding, photosystem II, light-harvesting complex
Family: across families, mostly in PF00124 Photosynthetic reaction center protein
Transmembrane protein abundance: low, **Transmembrane protein specificity**: medium
No of hits in Swiss-Prot: 68
No of hits in Swiss-Prot-TM: 56
No of false positives: 12 (17.6%)
Interactions: different lipid binding sites, such as cardiolipins (modulators of protein function)
Mutations: no

WebLogo
Length 9

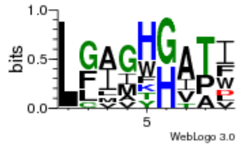

Structural Alignment

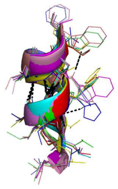

Hydrogen Bonds Pattern
Length 9

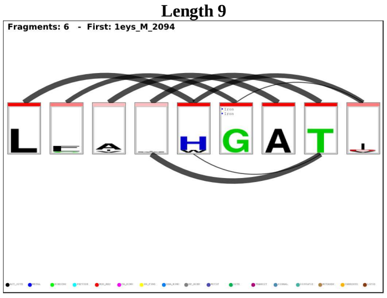

Backbone Torsion Angles
Length 9

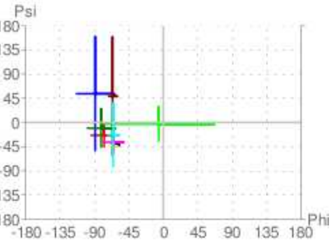

Cross-family helix-helix packing motif P-[ATV]-V-[QR]-x-G-[GL]-[NQR]-x-G

Structural characterisation: membrane-embedded tilted alpha-helix
Function: cytochrome-c oxidase activity, voltage-gated chloride channel activity
Family: across families, mostly in PF00510 Cytochrome c oxidase subunit III and PF00654 Voltage gated chloride channel
Transmembrane protein abundance: low, **Transmembrane protein specificity**: meidum
No of hits in Swiss-Prot: 49
No of hitsin Swiss-Prot-TM: 44
No of false positives: 5 (10%)
Interactions: protein-protein interaction interface
Mutations: no

WebLogo
Length 11

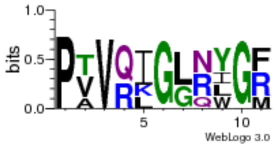

Structural Alignment

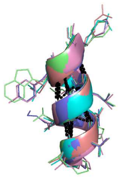

Hydrogen Bonds Pattern
Length 11

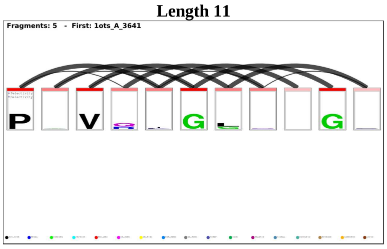

Backbone Torsion Angles
Length 11

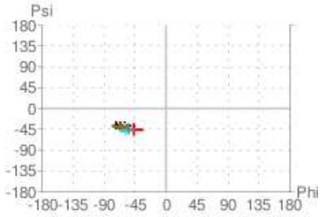

Heme binding pocket motif in cytochrome b R-x-[ILM]-H-A-N-G-A-S-[FLM]-F

Structural characterisation: regular helix motif at the end of alpha-helices
Function: iron ion binding, oxidoreductase activity, electron transport
Family: PF00033 Cytochrome b
Transmembrane protein abundance: high, **Transmembrane protein specificity**: high
No of hits in Swiss-Prot: 1504
No of hits in Swiss-Prot-TM: 1503
No of false positives: 21 (0.07%)
Interactions: heme binding, iron ion binding
Mutations: no

WebLogo
Length 13

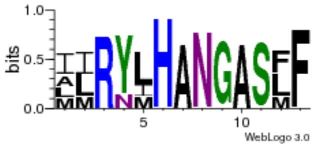

Structural Alignment

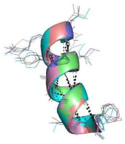

Hydrogen Bonds Pattern
Length 13

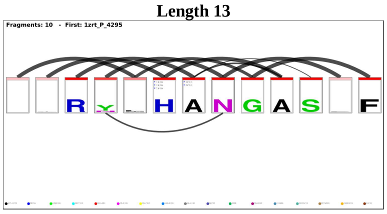

Backbone Torsion Angles
Length 13

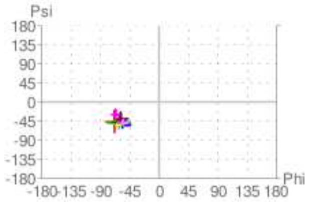

Cross-family cofactor binding motif G-P-x-[FY]-x-G-x(2)-G

Structural characterisation: irregular structure at the membrane-water interface
Function: loss of catalytic activity upon mutation of G and Y
Family: across families, mostly PF00146 NADH-dehydrogenase
Transmembrane protein abundance: medium, **Transmembrane protein specificity**: medium
No of hits in Swiss-Prot: 247
No of hits in Swiss-Prot-TM: 166
No of false positives: 81 (32.8%)
Interactions: cofactor binding
Mutations: no

WebLogo
Length 13

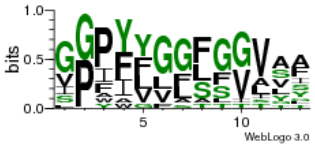

Structural Alignment

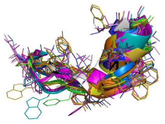

Hydrogen Bonds Pattern
Length 13

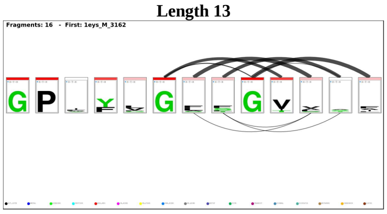

Backbone Torsion Angles
Length 13

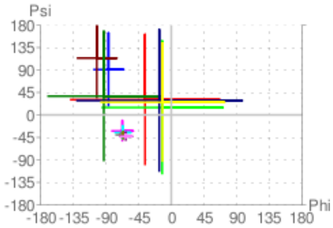

Glycerol binding site motif in bacteriorhodopsins Y-[LV]-[ASV]-M-x-[FL]-G-x-G-x-[TV]

Structural characterisation: irregular structure at the membrane-water interface
Function: photoreceptor activity, hydrogen ion transporter activity, ion channel activity
Family: PF01036 Bacteriorhodopsin
Transmembrane protein abundance: low, **Transmembrane protein specificity**: high
No of hits in Swiss-Prot: 13
No of hits in Swiss-Prot-TM: 13
No of false positives: 0 (0%)
Interactions: glycerol binding
Mutations: no

WebLogo
Length 13

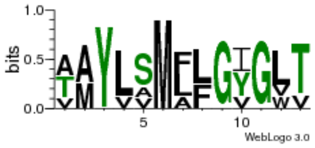

Structural Alignment

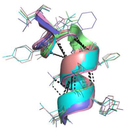

Hydrogen Bonds Pattern
Length 13

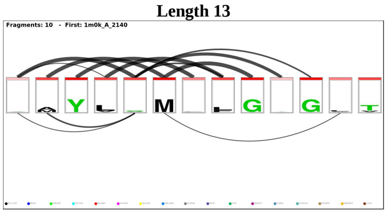

Backbone Torsion Angles
Length 13

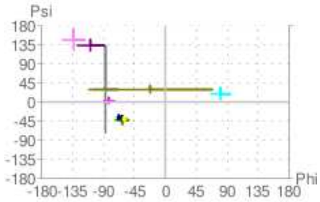

Putative lipid binding helical motif across families F-A-x-F-L-[FIV]-[FI]-[ILV]

Structural characterisation: membrane-embedded alpha-helix
Function: phosphate binding, photosystem II, mitochondrial electron transport chain
Family: across four distinct families, CL0062 APC superfamily, PF00737 Photosystem II, CL0111 Glycosyl transferase GT-C superfamily, PF00510 cytochrome c oxidase
Transmembrane protein abundance: low, **Transmembrane protein specificity**: high
No of hits in Swiss-Prot: 38
No of hits in Swiss-Prot-TM: 38
No of false positives: 0 (0%)
Interactions: lipid binding, heme binding
Mutations: no

WebLogo
Length 8

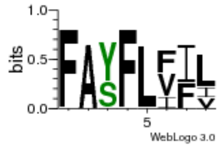

Structural Alignment

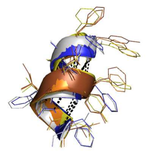

Hydrogen Bonds Pattern
Length 8

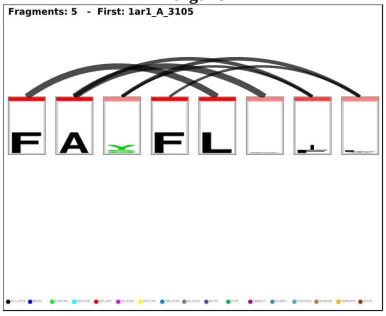

Backbone Torsion Angles
Length 8

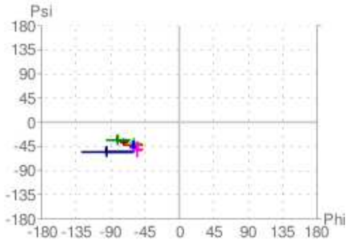

Length 9

Chlorophyll binding motif in photosystem I F-[LV]-G-x(0,2)-F-x(1,3)-G-[AL]-[FY]-V

Structural characterisation: membrane-embedded bent alpha-helix
Function: chloroplast, photosynthesis, metal ion binding, light-harvesting
Family: across families, mostly PF00223 Photosystem I
Transmembrane protein abundance: medium, **Transmembrane protein specificity**: high
No of hits in Swiss-Prot: 126
No of hits in Swiss-Prot-TM: 126
No of false positives: 0 (0%)
Interactions: chlorophyll binding, protein-protein interaction interface
Mutations: no

WebLogo
Length 13

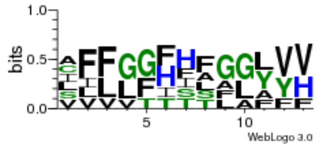

Structural Alignment

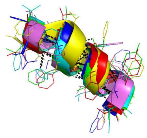

Hydrogen Bonds Pattern
Length 13

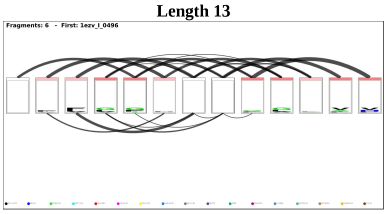

Backbone Torsion Angles
Length 13

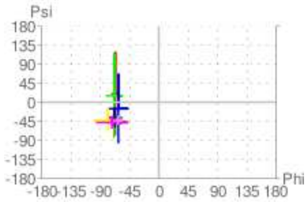

Across families loop motif G-A-G-[FIV]-[IV]-[GP]-[LM]-[EGN]-I-x(0,1)-E

Structural characterisation: irregular helix/loop at the membrane-water interface
Function: appears across more than one family binding, light-harvesting
Family: across families, mostly CL0192 G protein-coupled receptor-like superfamily
Transmembrane protein abundance: low, **Transmembrane protein specificity**: high
No of hits in Swiss-Prot: 8
No of hits in Swiss-Prot-TM: 8
No of false positives: 0 (0%)
Interactions: protein-protein interaction interface
Mutations: no

WebLogo
Length 11

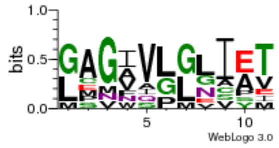

Structural Alignment

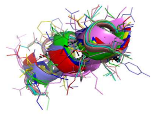

Hydrogen Bonds Pattern
Length 11

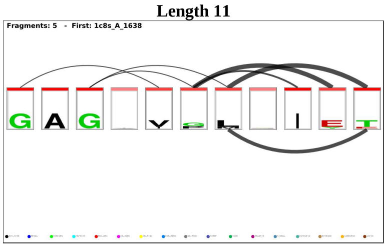

Backbone Torsion Angles
Length 11

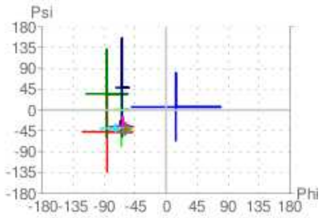

Water-membrane interface motif in cytochrome c and quinol oxidases G-G-F-G-N-[WY]-[FL]-[MV]

Structural characterisation: irregular 3/10 helix at the membrane-water interface
Function: metal ion binding, cytochrome-c oxidase activity, mitochondrial electron transport chain, aerobic respiration
Family: PF00115 cytochrome c and quinol oxidases
Transmembrane protein abundance: medium, **Transmembrane protein specificity**: high
No of hits in Swiss-Prot: 122
No of hits in Swiss-Prot-TM: 122
No of false positives: 0 (0%)
Interactions: protein-protein interaction interface
Mutations: no

WebLogo
Length 12

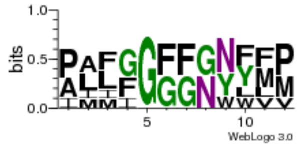

Structural Alignment

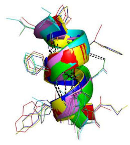

Hydrogen Bonds Pattern
Length 12

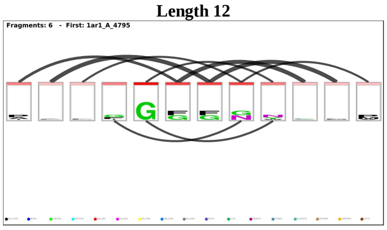

Backbone Torsion Angles
Length 12

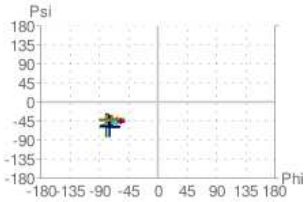

Chlorophyll binding loop motif in photosynthetic reaction center proteins L-x(1,2)-F-x(0,1)-R-P-[ILV]-[LM]-[LM]-G-[AS]-W

Structural characterisation: structured loop/beta strand connecting two alpha helices at the membrane-water interface
Function: bacteriochlorophyll binding, reaction center, light harvesting complex, photosynthesis
Family: PF00124 Photosynthetic reaction center protein
Transmembrane protein abundance: low, **Transmembrane protein specificity**: high
No of hits in Swiss-Prot: 10
No of hits in Swiss-Prot-TM: 10
No of false positives: 0 (0%)
Interactions: bacteriochlorophyll binding
Mutations: no

WebLogo
Length 12

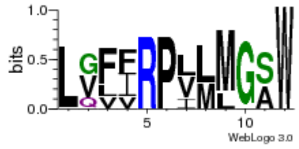

Structural Alignment

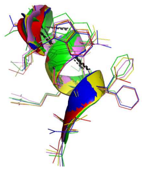

Hydrogen Bonds Pattern
Length 12

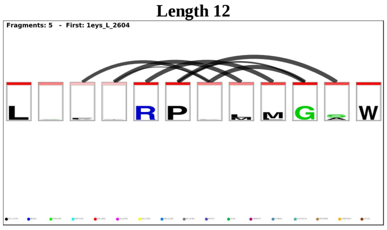

Backbone Torsion Angles
Length 12

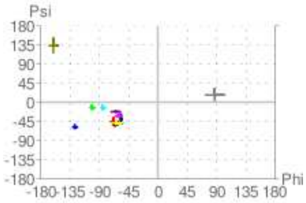

Heme binding loop motif in cytochrome b G-Y-[SV]-L-P-W

Structural characterisation: structured loop in the inter membrane space of mitochondria
Function: oxidoreductase activity, metal ion binidng, mitochondrial electron transport chain
Family: PF00033 Cytochrome b (N-terminal)
Transmembrane protein abundance: high, **Transmembrane protein specificity**: high
No of hits in Swiss-Prot: 1745
No of hits in Swiss-Prot-TM: 1744
No of false positives: 1 (0.06%)
Interactions: heme binding, stigmatellin A binding
Mutations: no

WebLogo
Length 10

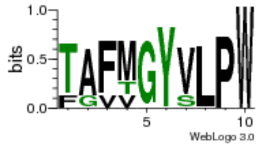

Structural Alignment

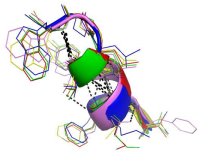

Hydrogen Bonds Pattern
Length 10

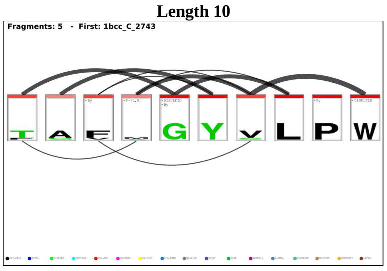

Backbone Torsion Angles
Length 10

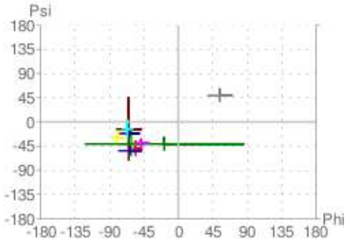

Retinal binding motif in G protein-coupled receptors L-[ALV]-F-[FM]-[AV]-x-D-V-x(2,3)-K-x(0,1)-G

Structural characterisation: bent alpha helix
Function: photoreceptor activity, Natch signaling pathway
Family: CL0192 G protein-coupled receptor-like superfamily
Transmembrane protein abundance: low, **Transmembrane protein specificity**: high
No of hits in Swiss-Prot: 20
No of hits in Swiss-Prot-TM: 20
No of false positives: 0 (0%)
Interactions: retinal binding
Mutations: no

WebLogo
Length 13

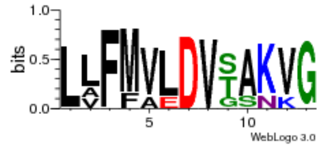

Structural Alignment

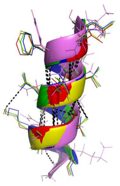

Hydrogen Bonds Pattern
Length 13

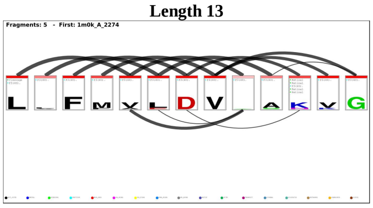

Backbone Torsion Angles
Length 13

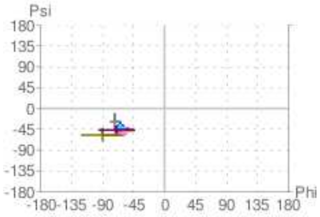

Carotene binding motif in photosynthetic reaction center proteins P-L-x-[DEK]-G-G-x-W-x(2)-[AI]-[GST]

Structural characterisation: irregular helix at the membrane-water interface
Function: photosynthesis, light reaction, electron transporter, light-harvesting complex
Family: CL0192 G protein-coupled receptor-like superfamily
Transmembrane protein abundance: low, **Transmembrane protein specificity**: high
No of hits in Swiss-Prot: 16
No of hits in Swiss-Prot-TM: 16
No of false positives: 0 (0%)
Interactions: carotene binding, aliphatic hydrocarbons binding
Mutations: no

WebLogo
Length 12

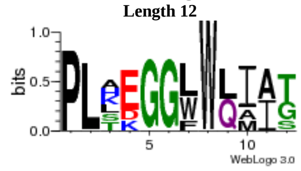

Structural Alignment

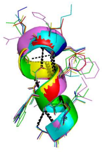

Hydrogen Bonds Pattern
Length 12

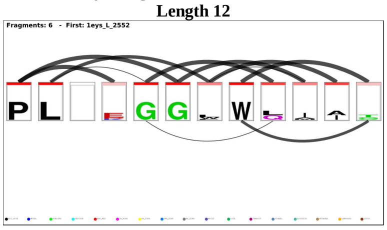

Backbone Torsion Angles
Length 12

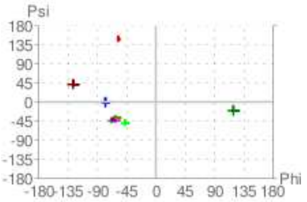

Tilted helical motif in cytochrome b W-x(0,1)-W-x(0,2)-G-x-[LV]-L-[AG]-[FILV]-[CT]-L

Structural characterisation: highly tilted helix with a 3/10 helical turn embedded into the membrane
Function: oxidoreductase activity, iron ion binding, mitochondrial electron transport chain
Family: PF00033 cytochrome b (N-terminal)
Transmembrane protein abundance: high, **Transmembrane protein specificity**: high
No of hits in Swiss-Prot: 1328
No of hits in Swiss-Prot-TM: 1327
No of false positives: 1 (0.07%)
Interactions: heme binding, ubiquinone binding, cardiolipin binding
Mutations: no

WebLogo
Length 12

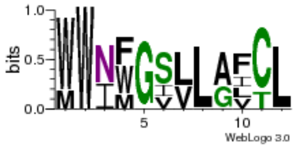

Structural Alignment

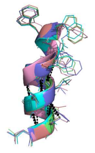

Hydrogen Bonds Pattern
Length 12

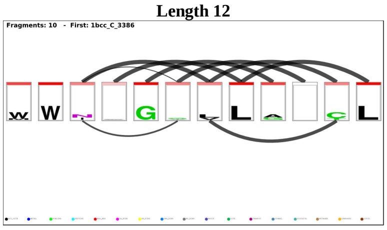

Backbone Torsion Angles
Length 12

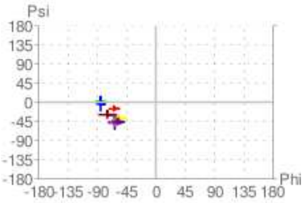

Proline-kink motif in cytochrome b H-[FY]-[IL]-[LV]-P-F-[AIV]-I-x(0,1)-A

Structural characterisation: membrane-embedded proline-kink in the middle of a halpha-helix
Function: oxidoreductase activity, iron ion binding, mitochondrial electron transport chain
Family: PF00033 cytochrome b (N-terminal)
Transmembrane protein abundance: high, **Transmembrane protein specificity**: high
No of hits in Swiss-Prot: 1083
No of hits in Swiss-Prot-TM: 1082
No of false positives: 1 (0.09%)
Interactions: iron binding (His residue), heme binding
Mutations: no

WebLogo
Length 10

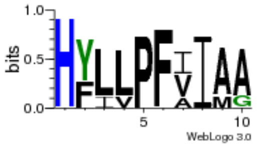

Structural Alignment

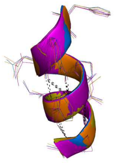

Hydrogen Bonds Pattern
Length 10

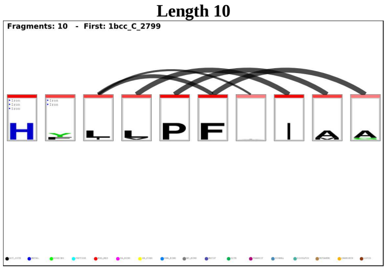

Backbone Torsion Angles
Length 10

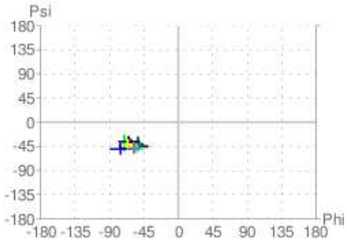

Interface helix motif in subunit III of bc1 complex G-L-Y-Y-G-S-Y

Structural characterisation: structured loop containing one helix turn connecting two alpha helices and located at the membrane-water interface
Function: oxidoreductase activity, iron ion binding, mitochondrial electron transport chain, important role in the assembly of the complex
Family: PF00033 cytochrome b (N-terminal)
Transmembrane protein abundance: high, **Transmembrane protein specificity**: high
No of hits in Swiss-Prot: 911
No of hits in Swiss-Prot-TM: 909
No of false positives: 2 (0.21%)
Interactions: heme binding, protein-protein interaction interface
Mutations: no

WebLogo
Length 10

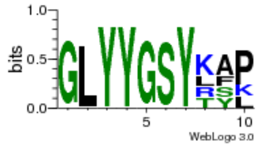

Structural Alignment

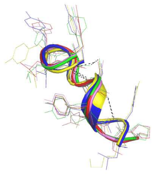

Hydrogen Bonds Pattern
Length 10

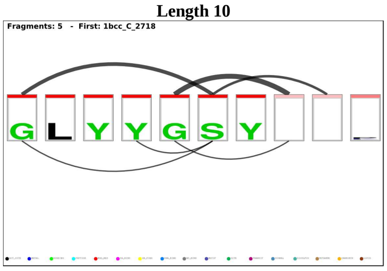

Backbone Torsion Angles
Length 10

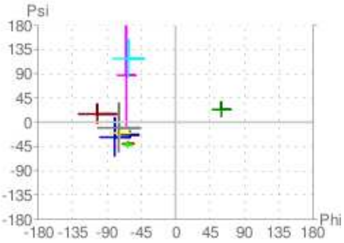

Bent helical motif at the membrane-water interface in cytochrome c and quinol oxidases I-L-G-A-[IV]-x(2)-[IP]-[TV]

Structural characterisation: bent terminal helix at the membrane-water interface
Function: oxidoreductase activity, iron ion binding, mitochondrial electron transport chain, important role in the assembly of the complex
Family: PF00115 cytochrome c and quinol oxidases
Transmembrane protein abundance: medium, **Transmembrane protein specificity**: high
No of hits in Swiss-Prot: 110
No of hits in Swiss-Prot-TM: 106
No of false positives: 4 (3.6%)
Interactions: protein-protein interaction interface
Mutations: no

WebLogo
Length 10

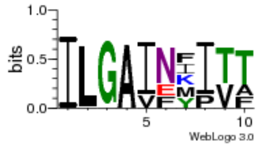

Structural Alignment

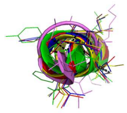

Hydrogen Bonds Pattern
Length 10

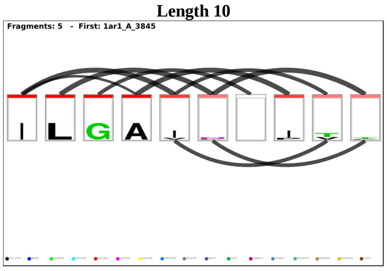

Backbone Torsion Angles
Length 10

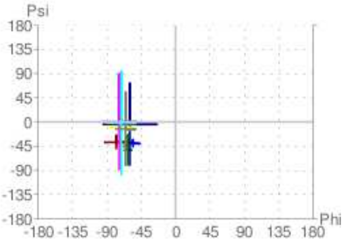

Structured heme-binding loop in heme-copper oxidases [WY]-x(2)-Y-P-P-L

Structural characterisation: structured loop in the membrane-water interface region, close to the extracellular side
Function: oxidoreductase activity, metal ion binding, mitochondrial electron transport chain
Family: PF00115 cytochrome c and quinol oxidases
Transmembrane protein abundance: medium, **Transmembrane protein specificity**: medium
No of hits in Swiss-Prot': 183
No of hits in Swiss-Prot-TM: 162
No of false positives: 21 (11%)
Interactions: heme binding
Mutations: no

WebLogo
Length 7

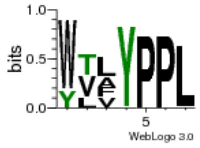

Structural Alignment

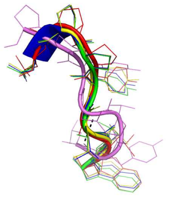

Hydrogen Bonds Pattern
Length 7
hydrogenbonds

Backbone Torsion Angles
Length 7

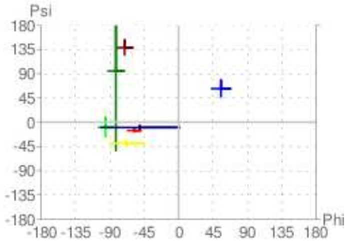

Supplement: Additional file 1 — List of 58 non-redundant significant novel motifs and their basic structural/functional descriptions. [file 1471-2105-11-204-S1.PDF]
