# Supplementary material for: Structural fragment clustering reveals novel structural and functional motifs in α-helical transmembrane proteins
Source: BMC Bioinformatics. 2010 Apr 26;11:204. doi: 10.1186/1471-2105-11-204 (PMC2876129; doi:10.1186/1471-2105-11-204)
Supplement: Additional file 2 — Statistics for both hydrogen bond- and backbone torsion angle-based clustering. [file 1471-2105-11-204-S2.PDF]

Supplementary 2 - Statistics for both hydrogen bonding and torsion angle clustering

For the torsion angle clustering, for each region but *Interface*, the number of fragments linearly decreases with the increasing fragment length (from 3 to 14 amino acids). An opposite trend is observed for fragments in the *Interface* region, as the longer the fragment the bigger the probability that such a fragment spans two regions (*Helix core* and either *Cytoplasm* or *Extracellular*) and it is therefore assigned to the *Interface* region from our method (see Fig. S2.1a). For the hydrogen bonding clustering the dependency of the number of fragments from the fragment size is not so obvious. The linearly increasing behaviour is still observed for the *Interface* region. For the *Helix core* region the number of fragments linearly decreases with the fragment size starting from size 4. For the *Cytoplasm*, *Extracellular* and *Reentrant* region the number of fragments increases with the fragment size up to a length of 6 amino acids and then remains constant (see Fig. S2.1b)

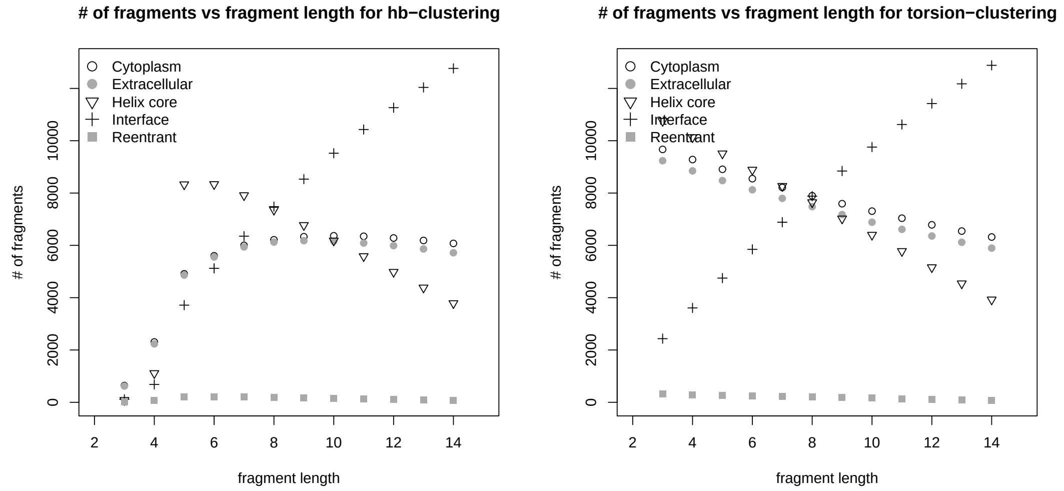

(a) # fragments vs fragment size for the hydrogen bonding

(b) # fragments vs fragment size for the torsion angle clustering

Figure S2.1: Dependency of number of fragments from the fragment size for both hydrogen bonding and torsion angle clustering.

The number of clusters varies in a non-linear fashion with the increasing fragment length for both hydrogen bonding and torsion angle clustering. The maximum number of clusters is obtained for fragments of size 14 in the *Interface* region (180) and size 10 in the *Cytoplasm* region (171) for torsion angle and hydrogen bonding clustering respectively (see Fig. S2.2a and Fig. S2.2b).

Also the number of outliers varies in a non-linear fashion with the fragment length and it is generally

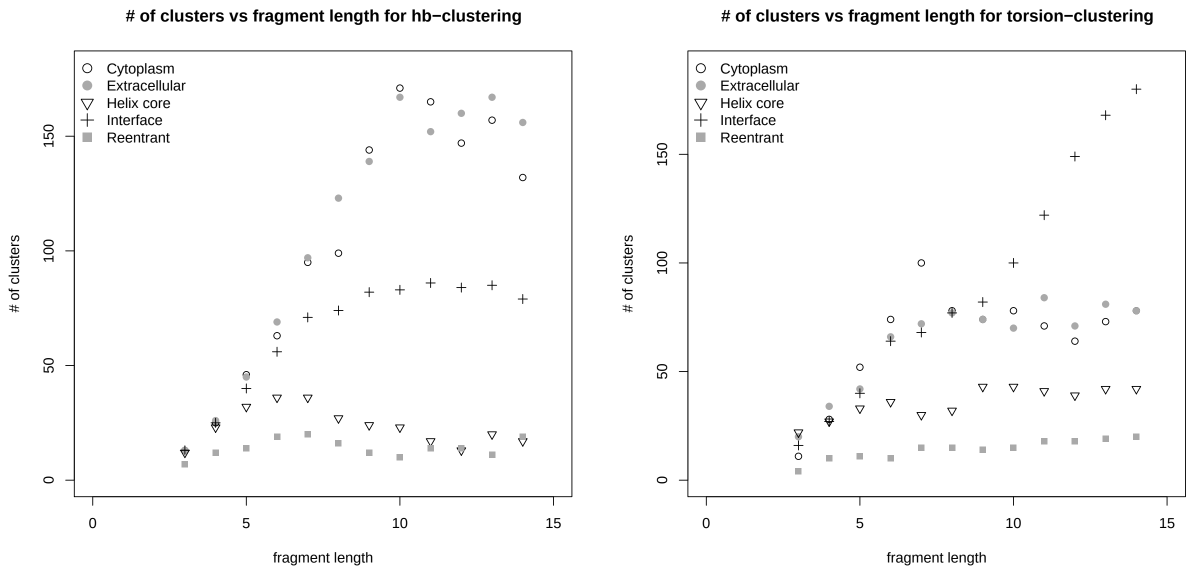

(a) # clusters vs fragment size for the hydrogen bonding clustering

(b) # clusters vs fragment size for the torsion angle clustering

Figure S2.2: Dependency of number of clusters from the fragment size for both hydrogen bonding and torsion angle clustering.

higher in the *Extracellular*, *Cytoplasm* and *Interface* regions with respect to the *Helix core* region (see Fig. S2.3a and Fig. S2.3b)

Fig. S2.4a and Fig. S2.4b show how the size of the largest cluster varies with the fragment length for both hb and torsion clustering. The largest cluster's size linearly decreases with the increasing fragment size foe the *Helix core* region, as expected, in both clustering. An opposite trend is observed for the *Interface* region. For the *Cytoplasm*, *Extracellular* and *Reentrant* regions the largest cluster's size is constant for fragments of length greater than 6 amino acids.

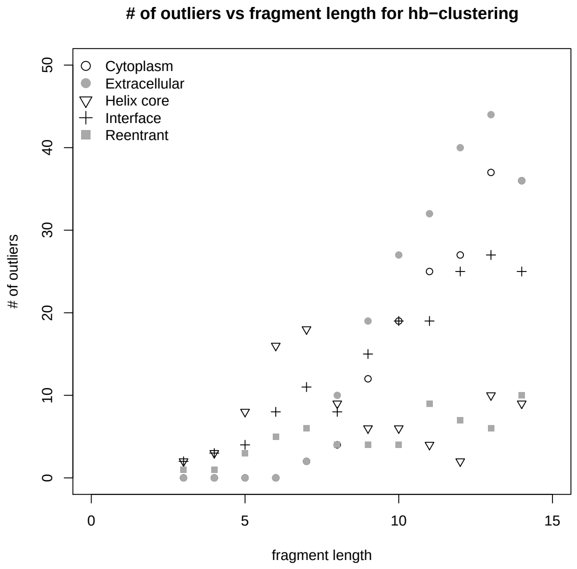

(a) # outliers vs fragment size for the hydrogen bonding clustering

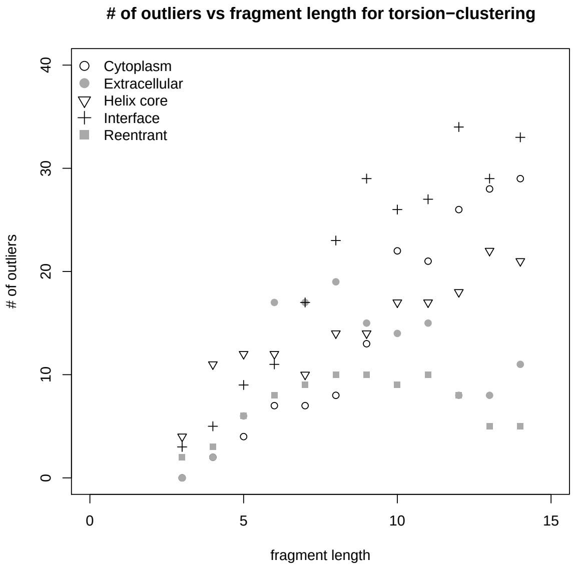

(b) # outliers vs fragment size for the torsion angle clustering

Figure S2.3: Dependency of number of outliers from the fragment size for both hydrogen bonding and torsion angle clustering.

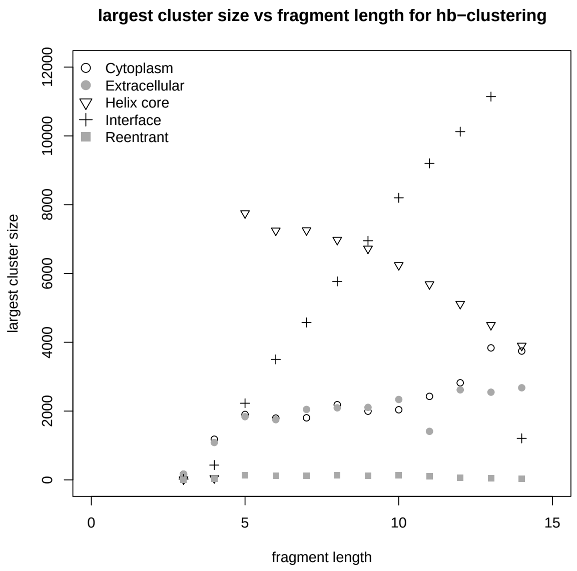

(a) largest cluster vs fragment size for the hydrogen bonding clustering

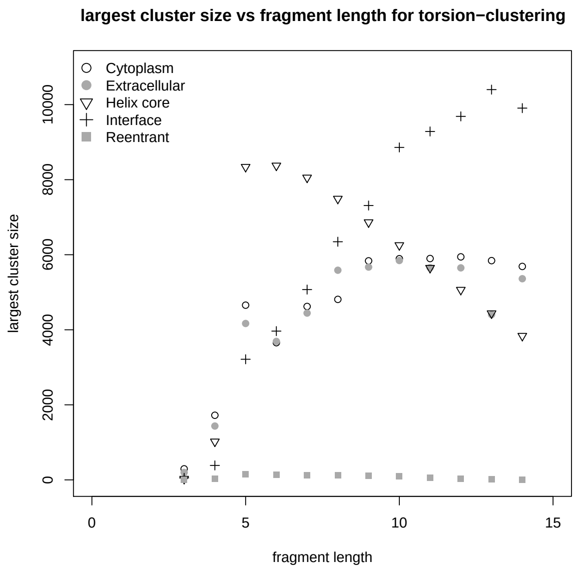

(b) largest cluster vs fragment size for the torsion angle clustering

Figure S2.4: Dependency of largest cluster's size from the fragment size for both hydrogen bonding and torsion angle clustering.
