# Supplementary material for: Structural fragment clustering reveals novel structural and functional motifs in α-helical transmembrane proteins
Source: BMC Bioinformatics. 2010 Apr 26;11:204. doi: 10.1186/1471-2105-11-204 (PMC2876129; doi:10.1186/1471-2105-11-204)
Supplement: Additional file 3 — Examples of very well known structural motifs: 310-helix and Schellmann motif. [file 1471-2105-11-204-S3.PDF]

Supplementary 3 - Examples of well known structural motifs: 3_{10}-helix and Schellmann motif

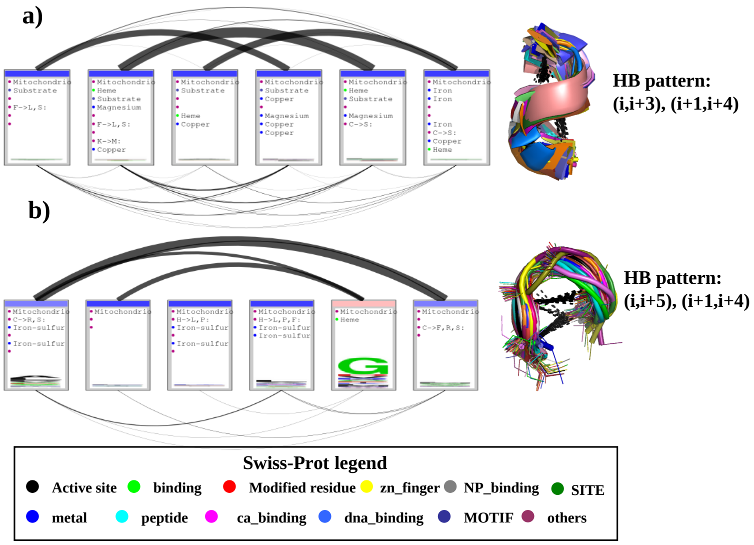

Figure S3: Two examples of very common structural motifs derived by both backbone torsion angle- and hydrogen bond-based clustering. Each rectangular box in the pictures corresponds to a given residue position in the fragment. Highly conserved hydrogen bond patterns between residues main chain atoms (upper arcs in the pictures) and side chain atoms (lower arcs in the picture) are shown by thicker arcs. Boxes corresponding to different residue positions are coloured on the basis of the conservation score of a residue inside a cluster, ranging from red (highly conserved) to blue (weakly conserved). Dots of different colours in the boxes correspond to the Features Swiss-Prot annotation according to the legend below. **a)** Motif corresponding to short 3_{10} helices, with the hydrogen bond pattern $(i, i+3)(i+1, i+4)$, derived from clustering of fragments of size 6 in the *Cytoplasm* region. 3_{10} helix motifs seem to be enriched in annotation relative to metal binding sites. **b)** Schellmann motif, with the typical hydrogen bond pattern $(i, i+5)(i+1, i+4)$ and a highly conserved glycine at position $i+4$ also derived from clustering of fragments of size 6 in the *Cytoplasm* region.
