# Supplementary material for: Structural fragment clustering reveals novel structural and functional motifs in α-helical transmembrane proteins
Source: BMC Bioinformatics. 2010 Apr 26;11:204. doi: 10.1186/1471-2105-11-204 (PMC2876129; doi:10.1186/1471-2105-11-204)
Supplement: Additional file 4 — Selection of the Prosite comparison cutoff. [file 1471-2105-11-204-S4.PDF]

Supplementary 4 - Selection of the prosite comparison cutoff

A justification for the choice of the 0.86 similarity score-cutoff for the comparison between our motif library and the Prosite-TM dataset (prosite patterns specific to transmembrane proteins) is given here. The fact that the choice of the cutoff has a dramatic effect on the statistics of the data strongly suggest that the choise must be based on a solid comparison to a random model. To this goal, a random model is constructed by randomizing the Prosite-TM dataset. The 125 motifs from the Prosite-TM dataset are joint into a string; the string is shuffled and random patterns, of lengths between the minimum and the maximum length in the original dataset, are generated. Patterns of different lengths are emitted according to the frequencies of occurrence of a given length in the original Prosite-TM dataset. Fig. S4 shows, respectively, the number of matches between our motif library and the Prosite-TM dataset (green line), and the number of matches between our motif library and the randomized Prosite-TM dataset (blue line). The cutoff of 0.86 is chosen because it maximizes the difference in number of matches (red line) between the randomzied Prosite-TM and the Prosite-TM dataset.

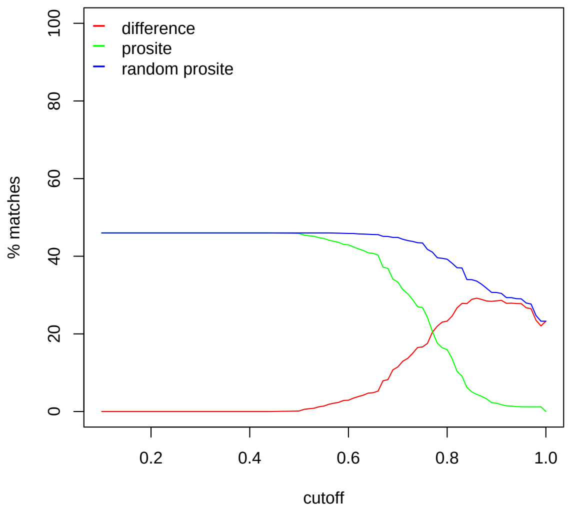

Figure S4: Comparison of the Prosite matching of our motif library with a random model obtained by shuffeling the Prosite-TM dataset. The green line corresponds to the number of matches between our motif library and the Prosite-TM dataset for each similarity score cutoff value in the range between 0.01 and 1.0; the blue line represents the percentage of matches between our motif library and the randomized Prosite-TM dataset and the red line the differences between the previous two sets. The maximum difference in the number of matches between Prosite and a random model is reached for a similarity cutoff of 0.86
