# Supplementary material for: Structural fragment clustering reveals novel structural and functional motifs in α-helical transmembrane proteins
Source: BMC Bioinformatics. 2010 Apr 26;11:204. doi: 10.1186/1471-2105-11-204 (PMC2876129; doi:10.1186/1471-2105-11-204)
Supplement: Additional file 5 — Distributions of minimum and average resolution of clusters. [file 1471-2105-11-204-S5.PDF]

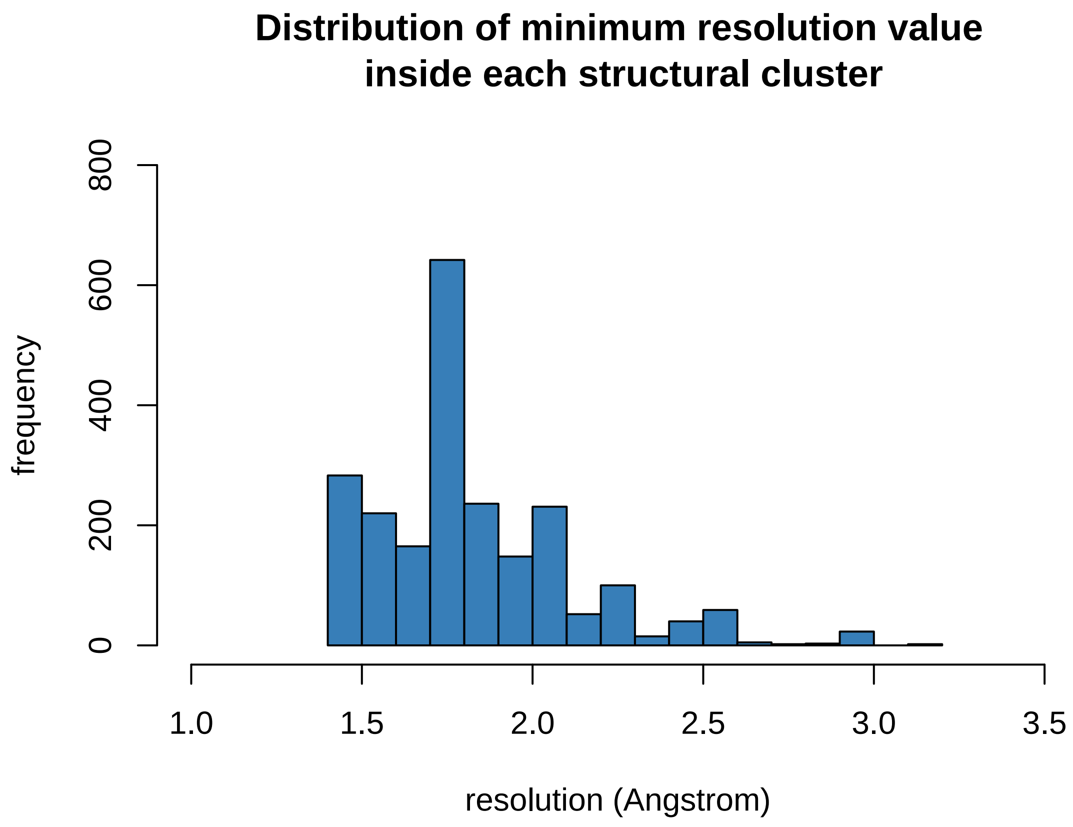
Distribution of minimum resolution value
inside each structural cluster
frequency
0
200
400
600
800
1.0
1.5
2.0
2.5
3.0
3.5
resolution (Angstrom)

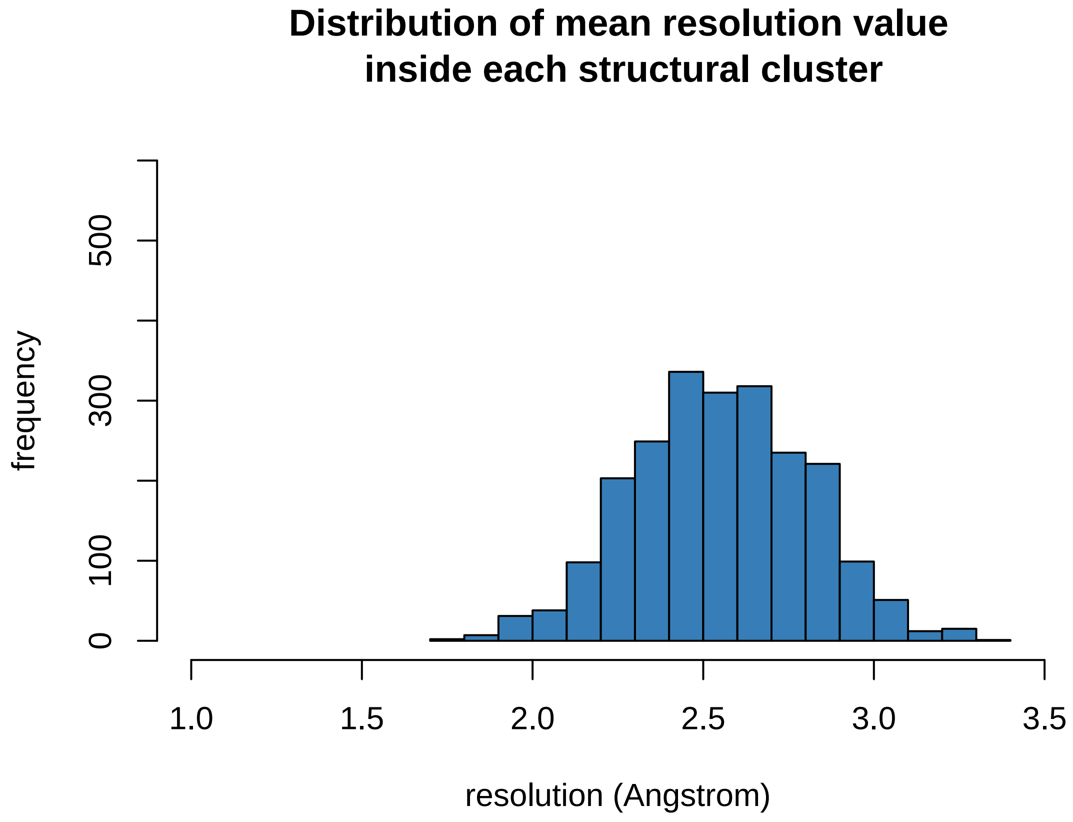
Distribution of mean resolution value
inside each structural cluster
frequency
0
100
300
500
1.0
1.5
2.0
2.5
3.0
3.5
resolution (Angstrom)
